# Supplementary material for: A Pseudomonas aeruginosa TIR effector mediates immune evasion by targeting UBAP1 and TLR adaptors
Source: EMBO J. 2017 May 8;36(13):1869–87. doi: 10.15252/embj.201695343 (PMC5494471; doi:10.15252/embj.201695343)
Supplement: Supplementary file 1 — Appendix [file EMBJ-36-1869-s001.pdf]

Table of content for the Appendix

Appendix Supplementary Figures

Figure S1
Figure S2
Figure S3
Figure S4
Figure S5

Appendix supplementary Figure Legends

A

Secondary structure of hMyD88

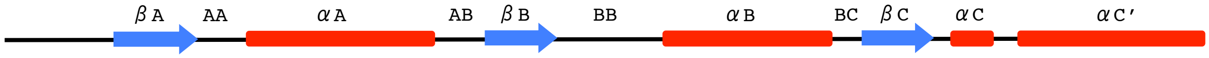


```
hMyD88-TIR  DDPLGHMPERFDAFICYCPSDIQ-FV-QEMIRQLEQTNYRLKLCVSDRDV--LPGTCVWSI-ASELIEKRCRRMVVVVSDDYLQSKECDFQTKFALSLSP
hTLR2-TIR   --------ICYDAFVSYSERDAY-WVENLMVQELENFNPPFKLCLHKRDF--IPGKWIIDN-IIDSIEK-SHKTVFVLSENFVKSEWCKYELDFSHFRLF
hTLR4-TIR   ----------YDAFVIYSSQDED-WVRNELVKNLEEGVPPFQLCLHYRDF--IPGVAIAANIIHEGFHK-SRKVIVVVSQHFIQSRWCIFEYEIAQTWQF
BtpA-TIR    -------EEEYDFFISHASEDKEAFV-QDLAAALRDL--GAKIFYDAYTL--KVGDSLRRK-IDQGLAN-SKFGIVVLSEHFFSKQWPARELDGLTAMEI
BtpB-TIR    ----------FDVGLSFPGEARG-LV-EQVARELEARVGPNAYFYDNNYVSQLARPSLDTL-LQDIYRNRCKLIVVFVGDDYQRKDWCGVEFRAIREIIM
TirS-TIR    ---------EYDVFLSHSSLDKEDYV-SKISEKLIEK--GLKVFEDVKVF--EIGKSQTET-MNMGILN-SRFVVVFLSPNFIESGWSRYEFLSFLNREI
PumA-TIR    ----------MAVFISYSHADKE--KIDMIAGHLVRK--RASVWVDRWEL--KPGDSLINR-IQEAVEG-SSALLIMLSSASVESEWCKKELTGGLLREL
```

Secondary structure of hMyD88

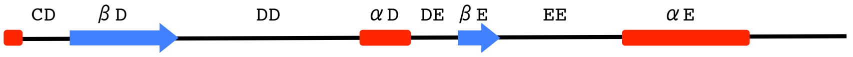


```
hMyD88-TIR  GAHQKRLIPIKYKAMKK---------EFPSILRFITVCDYTN--PCTKSWFWTRLAKALSLP---
hTLR2-TIR   DENNDAAILILLEPIEKKAIPQRFCKLRKIMNTKTYLEWPMD-EAQREGFWVNLRAAIKS----
hTLR4-TIR   LSSRAGIIFIVLQKVEKTLLRQQ-VELYRLLSRNTYLEWEDS-VLGRHIFWRRLRKALLDGKSW
BtpA-TIR    GG-QTRILPIWHKVSYDEVRRF-----SPSLADKVALNTSL----KSVEEIAKELHSLI-----
BtpB-TIR    ARAEQRIMFVRVDDGAVD-----------GVFRTDGYVDARRFNPSEIAQFIAERVALIT-----
TirS-TIR    NEEHVIILPIWHKVSVEDVRAY-----NPYLVDKYALNTSD---FSIEEIVEKIYQVIVNSKN-
PumA-TIR    EERRVVTIPVLLEDCK-----------IPLFLRDKLYADFRKDFDVGMSALLEAVAGHSNPDQSR
```

B

```
          10        20        30        40        50        60        70
MAVFISYSHADKEKIDMIAGHLVRKRASVWVDRWELKPGDSLINRIQEAVEGSSALLIMLSSASVESEWCKKELTGGLL
80        90        100       110       120       130       140       150
RELEERRVVTIPVLLEDCKIPLFLRDKLYADFRKDFDVGMSALLEAVAGHSNPDQSRIEDVDGYLDWATDWGEVEGNIS
 160       170       180       190       200       210       220       230
INYTLVQNSSNTEMTFLTQIFCILGSVASARYRQYQKLGIDWVYRTMHALSLQAFSKDNDDMFVILDDTIPKTRSLMCV
  240       250       260       270       280       290       300
DPKTGSDYEMKVICRKMGNDNGKDQLLNITEYLERIFEFTMKTNRKPTAEEEEKMKIIAATPWPRA
```

C

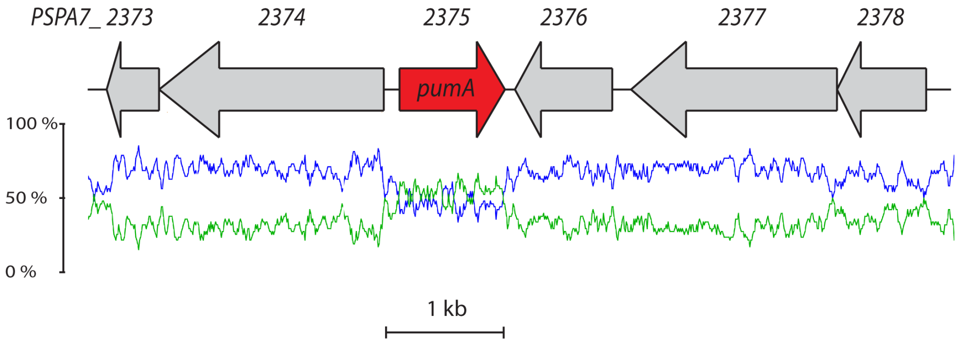

Appendix Figure S1

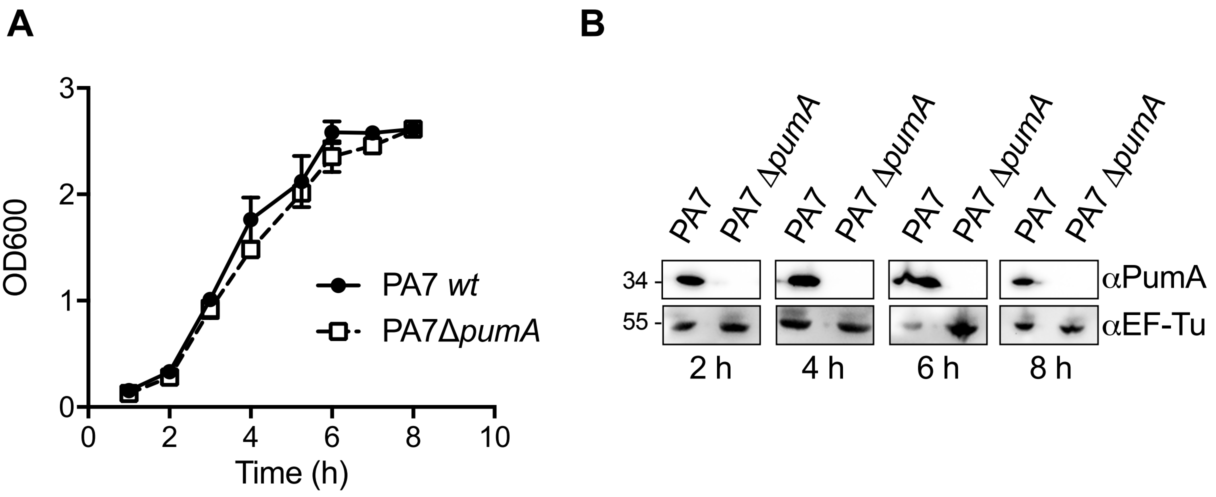

Appendix Figure S2

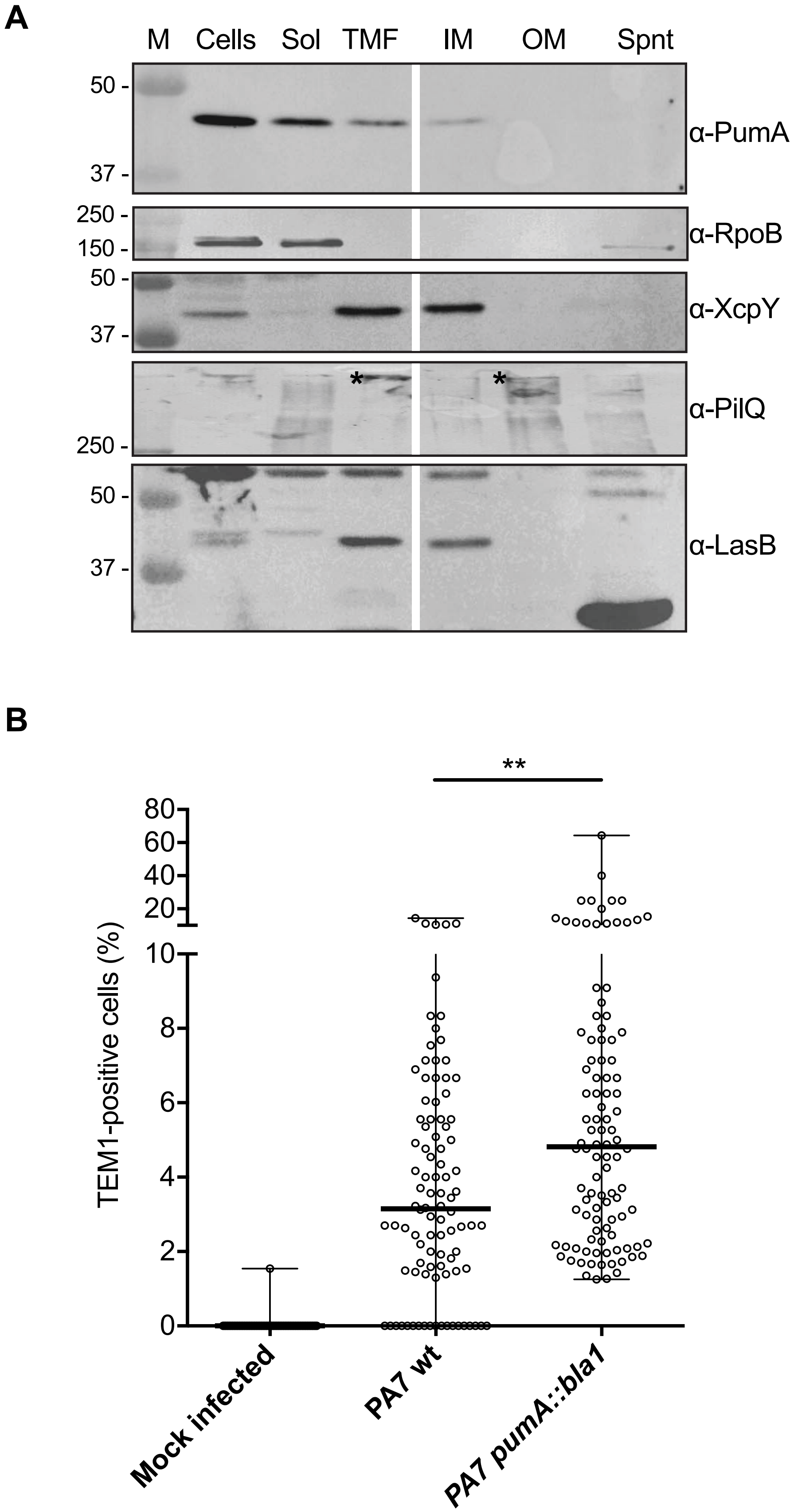

Appendix Figure S3

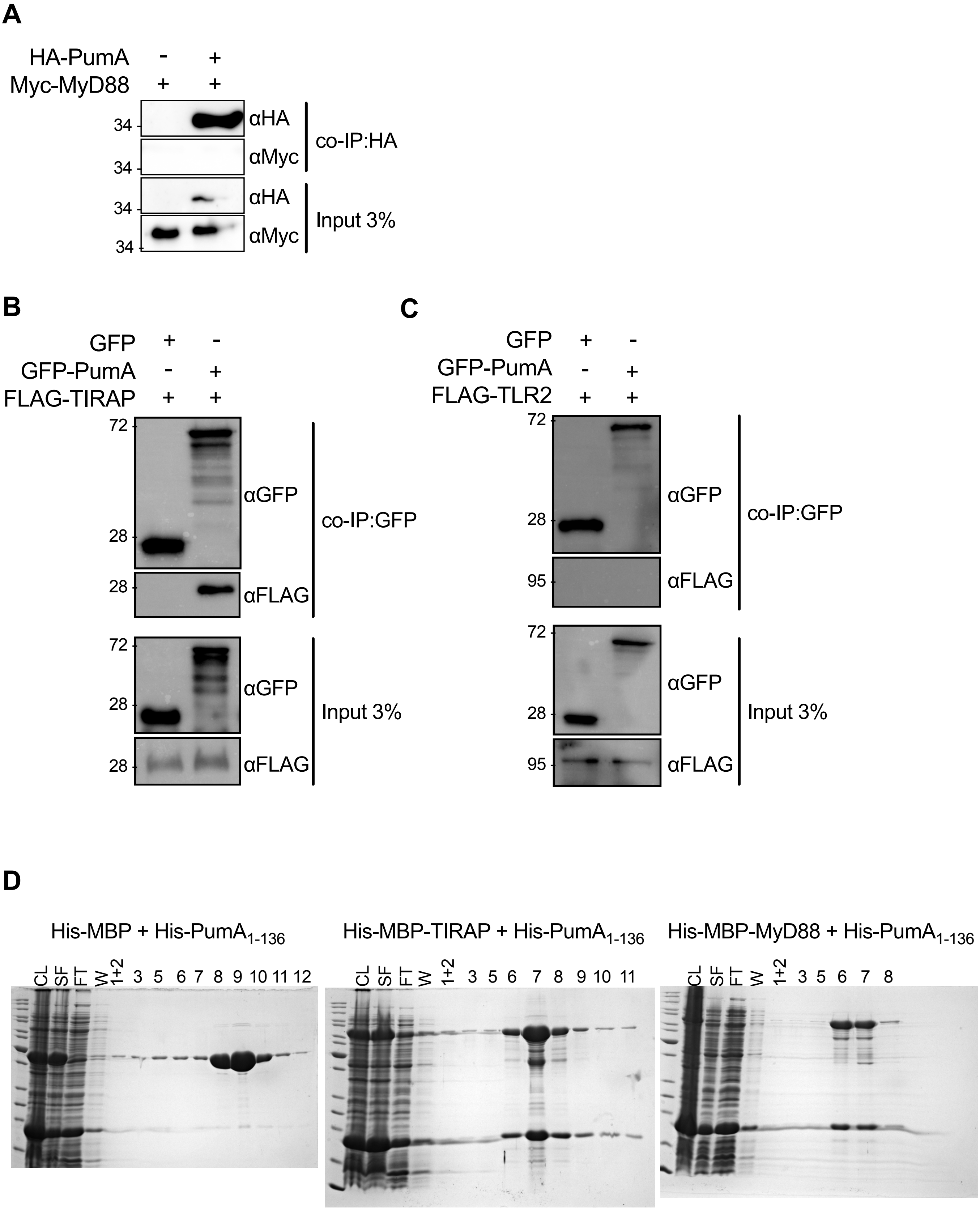

Appendix Figure S4

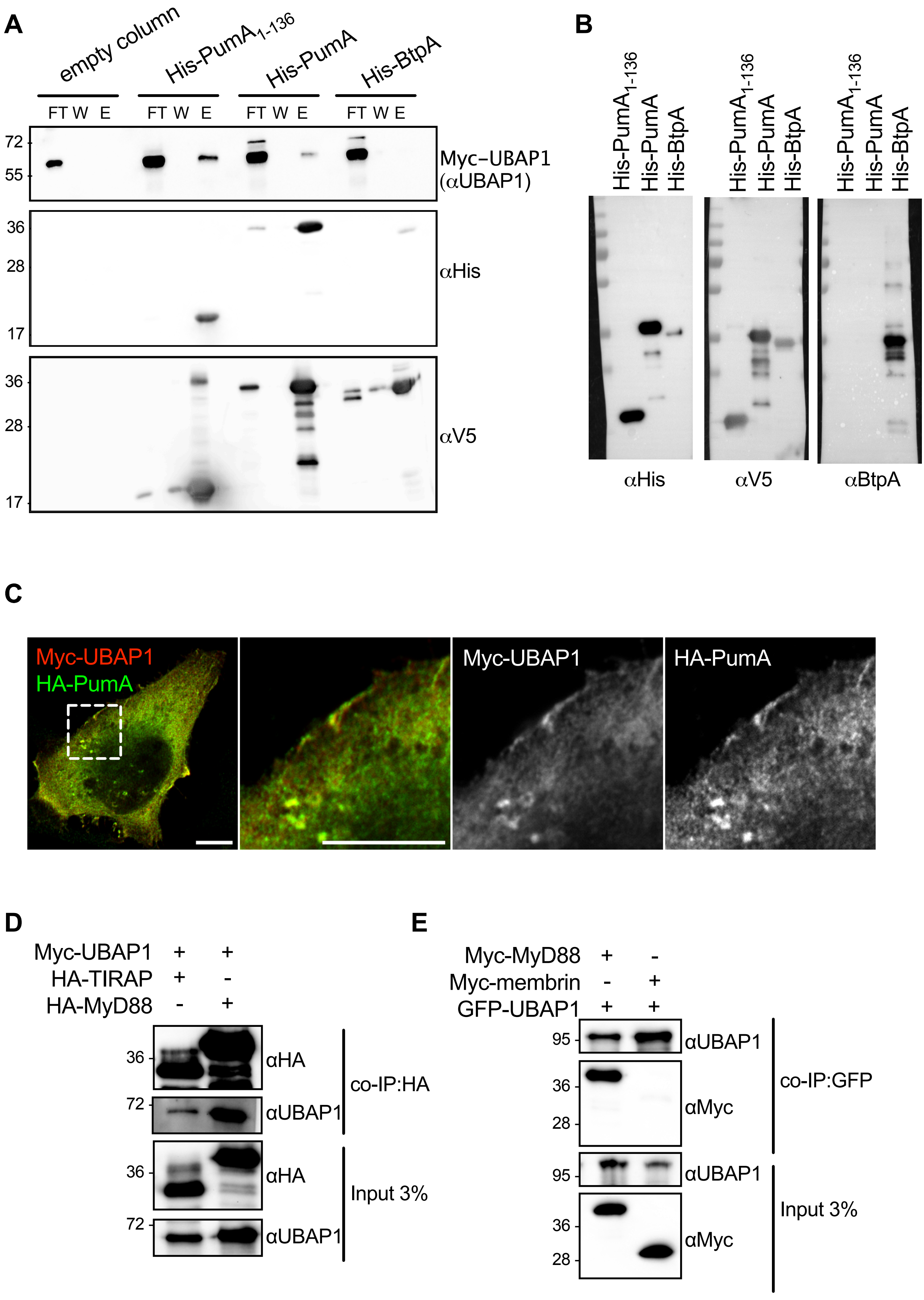

Appendix Figure S5

Appendix supplementary Figure Legends

Appendix Figure S1. Identification of a *Pseudomonas* TIR protein and analysis of its genetic context. (A) Multi-alignment of selected sequences of TIR domains from human and bacterial proteins for secondary structure prediction (PROMALS3D). The amino acid residues coloured in blue are predicted to be part of β-strands and in red of α-helixes. Secondary structures for hMyD88 are shown as blue arrows (β), red bars (α) and black line (connecting loops) above the sequences. (B) Full amino acid sequence of PumA (1-303). TIR domain is underlined in red. (C) G+C content determined using Geneious with a sliding window size of 50 bp. The blue graph represents G+C content while the green graph shows A+T content.

Appendix Figure S2. Analysis of PumA expression during growth. (A) Growth curve of *P. aeruginosa* PA7 wt and Δ*pumA* associated to (B) western blot of PumA production in liquid cultures. Native PumA was visualized using a polyclonal rabbit anti-PumA and band corresponds to 34 kDa. Control blot against the standard cytoplasmic protein EF-Tu (45 kDa) is shown below.

Appendix Figure S3. PumA is a cytoplasmic protein, translocated into host cells during infection. (A) Subcellular localisation of PumA was analysed by fractionation of PA7. PumA is mostly present in the soluble fraction and inner membrane. Integrity of the fractions was controlled by detection of RNA polymerase (RpoB) in the soluble fraction (Sol), T2SS inner membrane (IM) protein XcpY, the type IV pilus PilQ multimeric secretin in the outer membrane (OM) indicated by the asterisks and LasB exoprotein in the secreted fraction/supernatant (Spnt). The total membrane fraction was also included (TMF). The

molecular weight marker (M) is shown (kDa). (B) Translocation of PumA was observed by infecting A549 cells with wt PA7 versus a wt PA7 with a chromosomal PumA fusion with TEM1. At least 2500 cells were scored for each strain and graph corresponds to percentage of TEM1-positive cells from 6 independent experiments. Data corresponds to median ± range. Non-parametric One-way ANOVA Kruskal-Wallis test was performed, $P < 0.01$ denoted with **.

Appendix Figure S4. Additional co-IP experiments with PumA. (A) Co-IP from cells expressing HA-PumA and Myc-MyD88. The fractions bound to the HA-trapping beads were revealed with anti-Myc antibody. Western blot of the input is shown (anti-HA antibody). (B) Co-IP from cell extracts co-expressing GFP and FLAG-TIRAP or GFP-PumA and FLAG-TIRAP. (C) Co-IP from cell extracts co-expressing GFP and FLAG-TLR2 or GFP-PumA and FLAG-TLR2. For (B) and (C), the fractions bound to the GFP-trapping beads were revealed with anti-FLAG antibody. Western blots of the input are shown (anti-GFP and -FLAG antibodies). (D) Co-purification of His-PumA$_{1\text{-}136}$ co-expressed in *E. coli* BL21 with either His-MBP (left panel), His-MBP-TIRAP (center panel) or His-MBP-MyD88 (right panel). Interactions were visualized with coomassie blue stained gels. Cell lysate (CL), soluble fraction (SF), flow-through (FT), wash (W) and elution fractions are shown for each sample and the size ladder included on the first line of each gel. The fractions corresponding to the elution peaks are: for His-PumA136/His-MBP fractions 8-9-10, for His-PumA136/His-MBP-TIRAP fractions 6-7-8-9 and for His-PumA136/His-MBP-Myd88 fractions 6-7.

Appendix Figure S5. Control pull-down for interaction of PumA with UBAP1 and western blots for detection of BtpA. (A) Pull-down assay using extracts from cells expressing Myc-UBAP1 against His-PumA or His-PumA$_{1\text{-}136}$ immobilized on a Ni NTA resin.

Empty column was used as a control for non-specific binding. Interactions were visualized by western blotting using anti-UBAP1 antibody, and column binding with anti-His (middle blot), followed by anti-V5 (lower blot). Non-bound fraction (FT), last wash (W) and elution (E) are shown for each sample and the molecular weights indicated (kDa). (B) Western blots of 1 µg of purified His-PumA$_{1-136}$, His-PumA or His-BtpA and revealed with anti-His and anti-V5 antibodies, following successive stripping events. (C) Representative confocal microscopy image of HeLa cells expressing Myc-UBAP1 (red) and HA-PumA (green). Scale bar corresponds to 10 µm. (D) Co-IP from cells expressing Myc-UBAP1 and either HA-TIRAP or HA-MyD88. (E) Co-IP from cells expressing GFP-UBAP1 and Myc-MyD88. In parallel, a cell extract from co-expression of GFP-UBAP1 and Myc-Membrin was used as a negative control for non-specific binding. Both co-IPs (D and E) were revealed using an anti-UBAP1 antibody, the fractions bound to either GFP- or HA-trapping beads using an anti-Myc or -HA antibody, respectively and the inputs using anti-Myc, -HA or -UBPA1 antibodies as indicated.
