# Supplementary material for: A Pseudomonas aeruginosa TIR effector mediates immune evasion by targeting UBAP1 and TLR adaptors
Source: EMBO J. 2017 May 8;36(13):1869–87. doi: 10.15252/embj.201695343 (PMC5494471; doi:10.15252/embj.201695343)
Supplement: Supplementary file 2 — Expanded View Figures PDF [file EMBJ-36-1869-s002.pdf]

Expanded View Figures

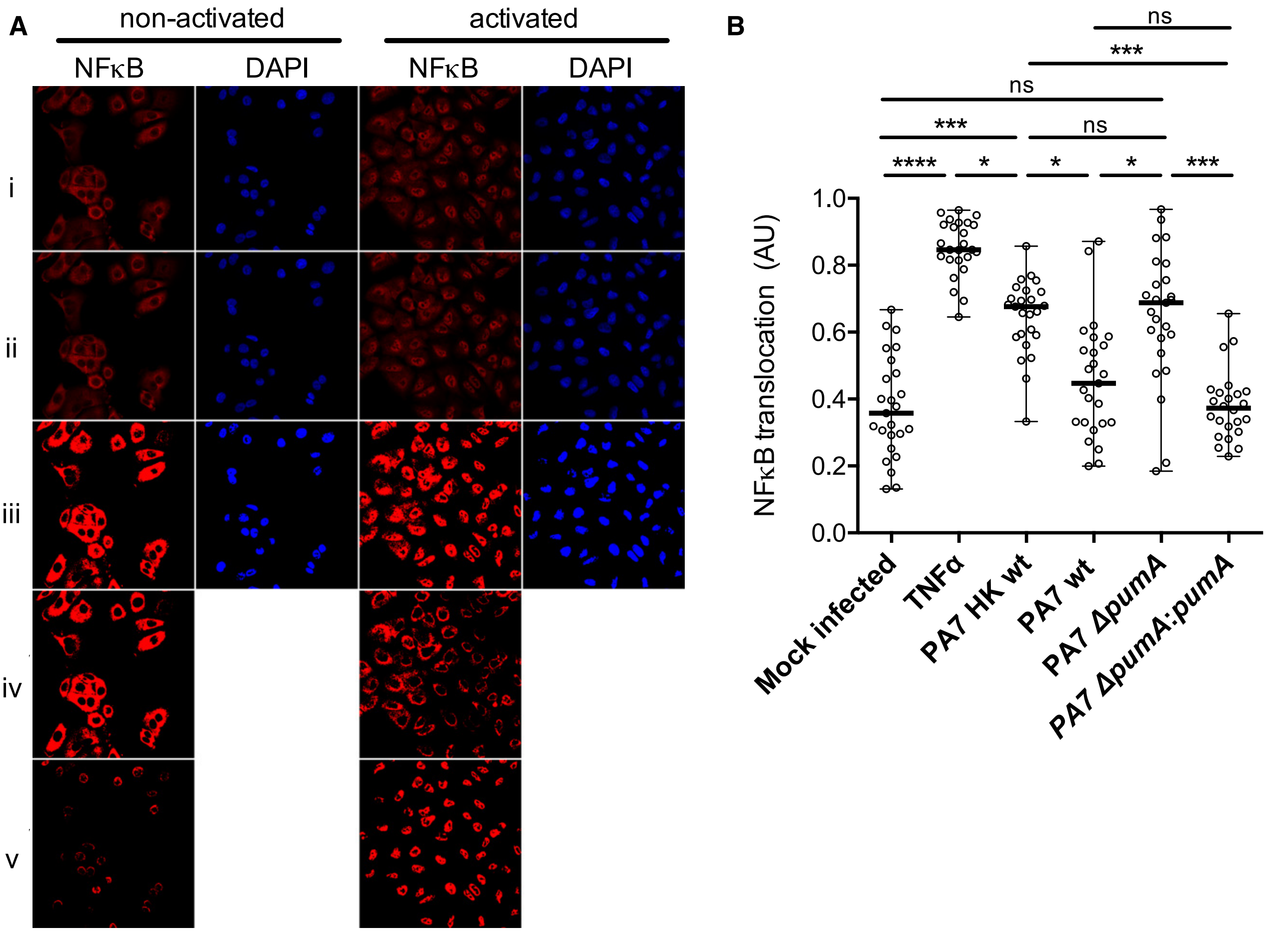

Figure EV1. Analysis of effect of *Pseudomonas* on NF-κB translocation into the nucleus.

A Images of treated/infected A549 cells were taken by confocal microscopy and analysed with a specific plugin developed in our team. NF-κB was labelled with an anti-p65 antibody (red) and nucleus with DAPI (blue). Rows correspond to (i) raw images, (ii) after median filter, (iii) treated with threshold moments, (iv) after subtraction of total NF-κB from DAPI to obtain the cytoplasmic NF-κB and (v) after subtraction of total NF-κB from NF-κB cytoplasmic to obtain nuclear NF-κB.

B Control experiment in which A549 cells were either mock infected, treated with TNFα (1 μg/ml) or infected with either heat-killed (HK) *P. aeruginosa* PA7, wt, Δ*pumA*, Δ*pumA::pumA* (Ara) induced with 1% arabinose. After 1 h cells were fixed, labelled as described in Materials and Methods, images taken by confocal microscopy and analysed with the plugin. Between 200 and 400 cells per condition were counted and data correspond to median ± standard error from three independent experiments. Non-parametric, one-way ANOVA, Kruskal–Wallis test was performed, with Dunn's multiple comparisons test, *****P* < 0.0001, ****P* < 0.001, **P* < 0.05.

 © 2017 The Authors

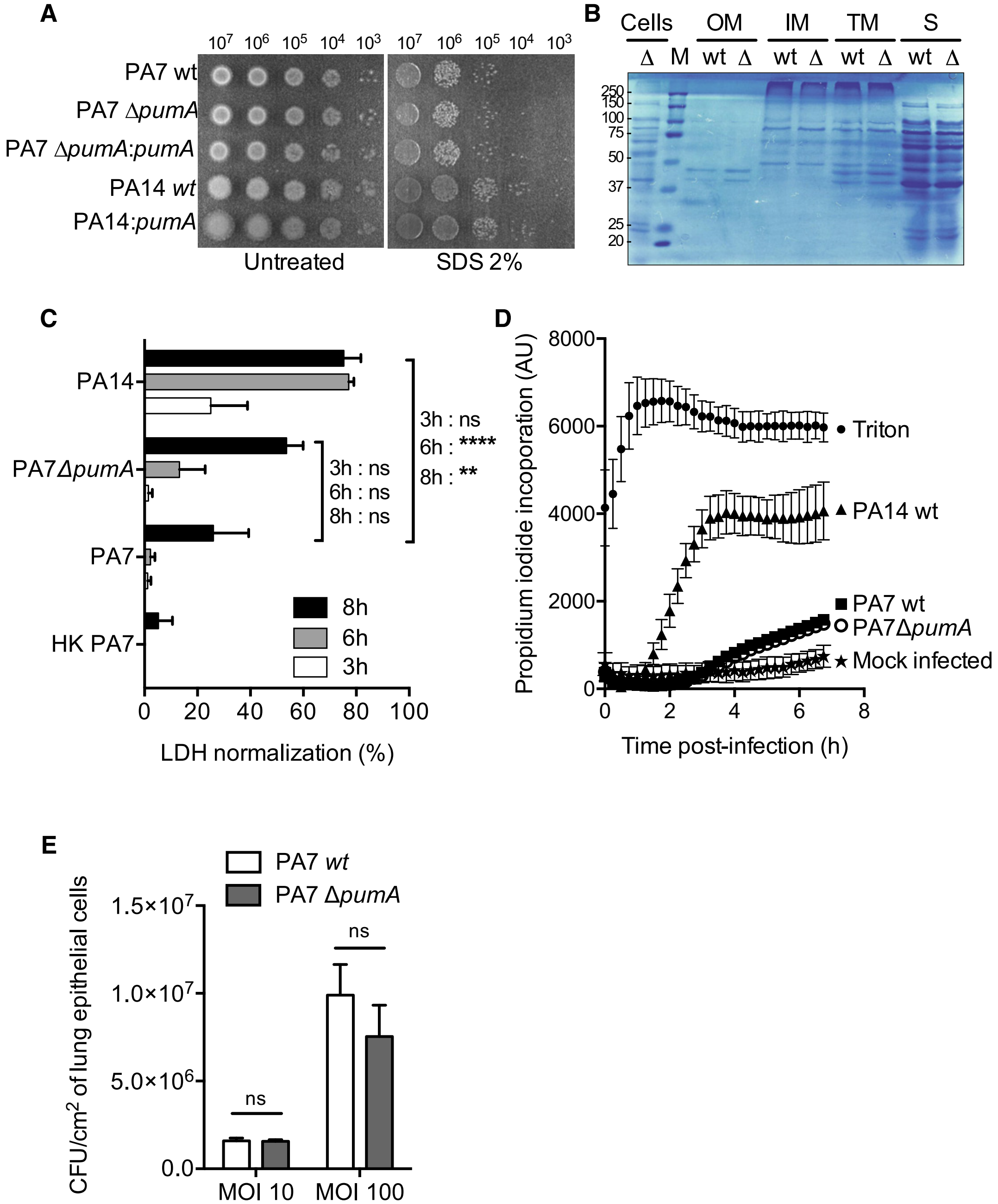

Figure EV2. PumA does not interfere with outer membrane stability and is not implicated in *P. aeruginosa* PA7 adhesion to host cells nor induction of cytotoxicity.

A Membrane permeability assay of the different *P. aeruginosa* strains in the presence or absence of 2% SDS.

B Analysis of total protein content of bacterial subcellular fractions through Coomassie staining of a polyacrylamide gel to visualize protein composition of the outer membrane (OM), inner membrane (IM), total membrane (TM) and secreted (S) fractions of PA7 compared to those of PA7 Δ*pumA*.

C LDH assay to measure cytotoxicity at 3, 6 and 8 h after treatment with TNFα or infection with PA7 wt, PA7 Δ*pumA*, PA14 wt and heat-killed PA7. Data correspond to means ± standard errors. A two-way ANOVA test was used, $***P < 0.0001$, $**P < 0.01$.

D Propidium iodide staining to measure cytotoxicity during 7 h of infection with PA7 wt, PA7 Δ*pumA* and PA14 wt as a positive control. Mock-infected sample corresponds to absorbance of non-infected cells treated in the same way as the infection and Triton corresponds to the maximum lysis. Data correspond to means ± standard errors from 3 independent experiments.

E Adhesion assay of PA7 wt and Δ*pumA* to A549 cells at MOI 10 and MOI 100. Non-parametric two-tailed Mann–Whitney test was performed, $P > 0.05$ denoted with ns. Data correspond to means ± standard errors from 3 independent experiments.

© 2017 The Authors

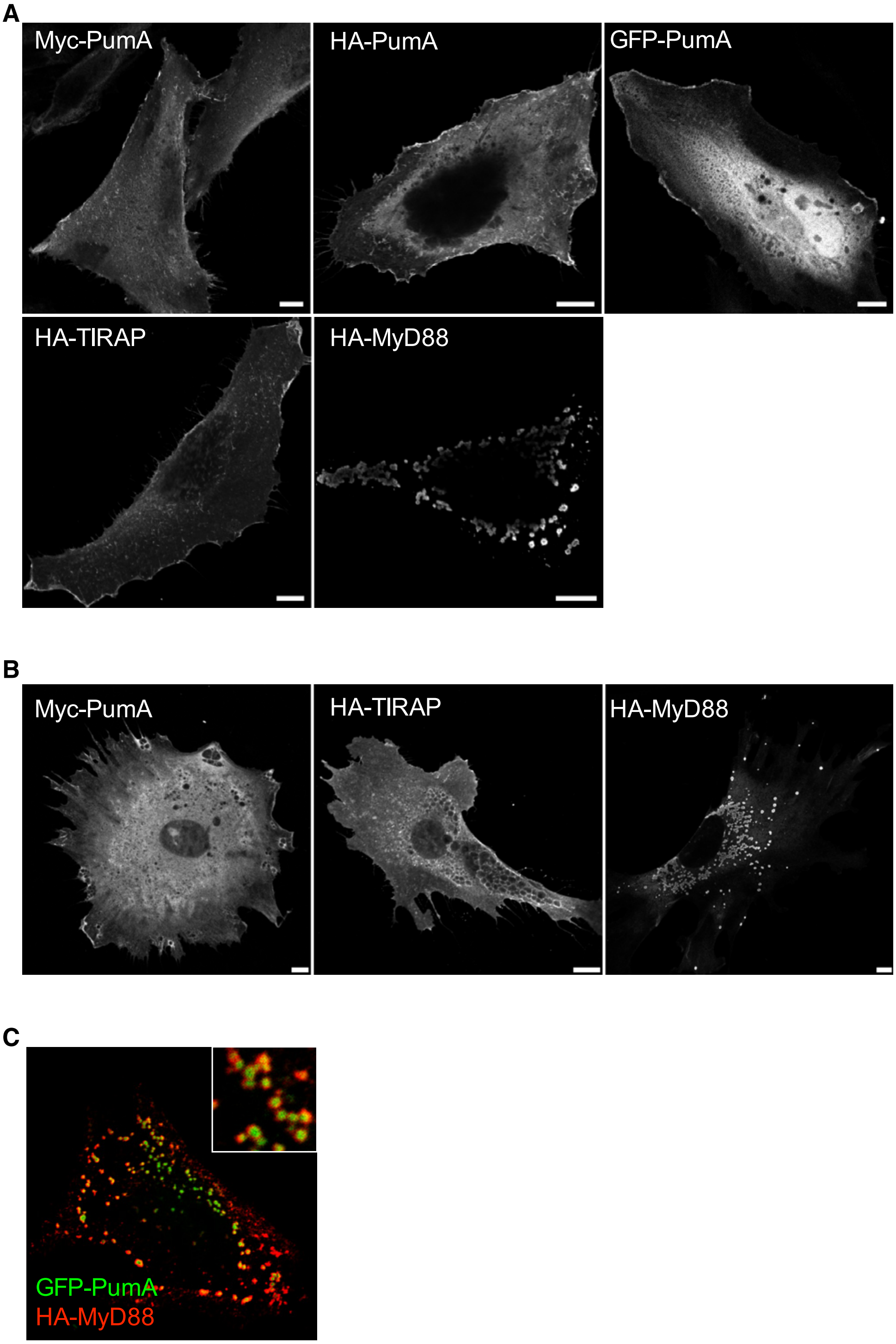

Figure EV3. Ectopic expression of PumA, TIRAP and MyD88 in HeLa and MEFs.

A Representative confocal immunofluorescence microscopy images of HeLa cells transfected with PumA fused to different tags or TIRAP or MyD88, fixed and labelled with the appropriate antibodies as indicated in each image. Scale bars correspond to 10 μm.

B Representative confocal immunofluorescence microscopy images of mouse embryonic fibroblasts (MEFs) transfected with Myc-PumA or HA-TIRAP or GFP-MyD88. Scale bars correspond to 10 μm.

C Confocal micrograph of a HeLa cell co-expressing GFP-PumA (green) and HA-MyD88 (red).

 © 2017 The Authors

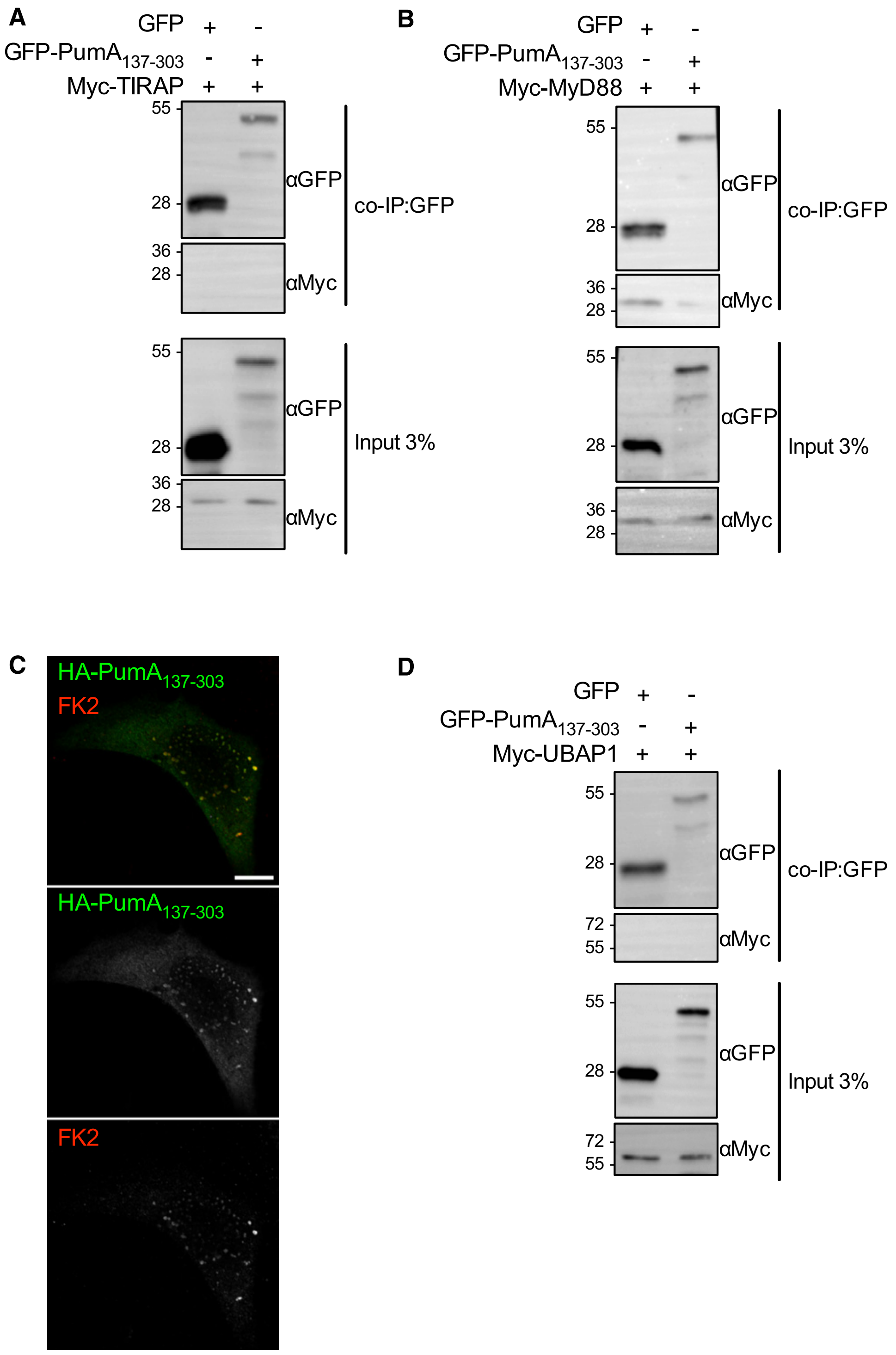

Figure EV4. Analysis of the role of the C-terminus of PumA.

A Co-IP from cell extracts co-expressing GFP and Myc-TIRAP or GFP-PumA$_{137–303}$ and Myc-TIRAP.

B Co-IP from cell extracts co-expressing GFP and Myc-MyD88 or GFP-PumA$_{137–303}$ and Myc-MyD88.

C Representative confocal image of cell expressing GFP-PumA$_{137–303}$ (green) labelled with FK2 antibody (red). Scale bar corresponds to 10 μm.

D Co-IP from cell extracts co-expressing GFP and Myc-UBAP1 or GFP-PumA$_{137–303}$ and Myc-UBAP1.

Data information: For all co-IPs, the fractions bound to the GFP-trapping beads were revealed with anti-Myc antibody. Western blots of the input are shown (anti-GFP and anti-Myc antibodies).

Source data are available online for this figure.

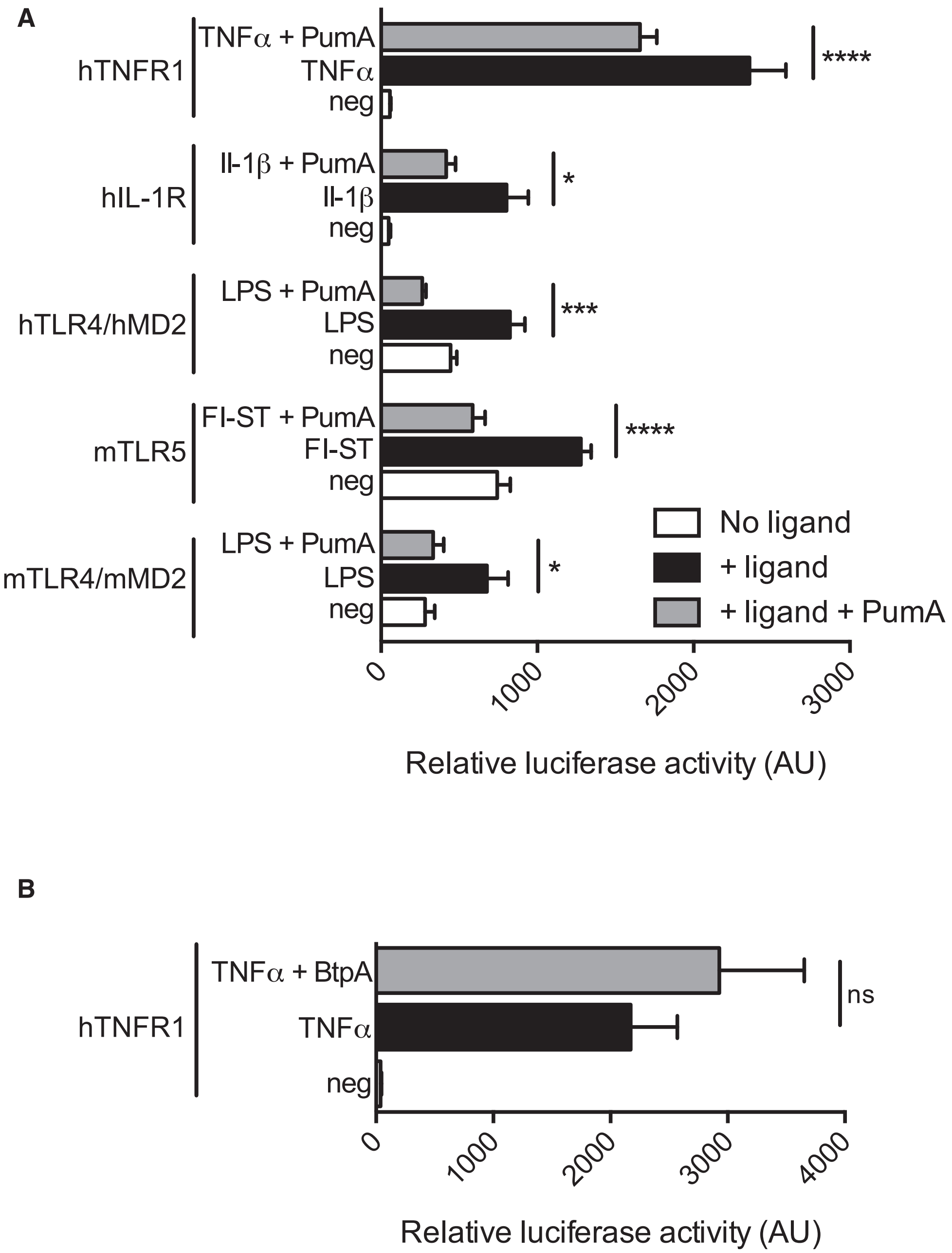

Figure EV5. PumA is able to reduce innate immune signalling *in vitro*.

A, B HEK 293T cells were transiently transfected with the NF-κB luciferase reporter vector in the presence or absence of plasmid (50 ng) encoding mouse/human TLRs and either (A) PumA or (B) BtpA/TcpB. In the case of human IL-1R and TNFR1, the endogenous receptors were induced by addition of the corresponding ligands. The total DNA amount was maintained constant between different conditions. After 24 h, cells were stimulated with the appropriate ligand, LPS, flagellin (Fl-ST), IL-1β or TNFα for 6 h and the luciferase activity was measured. All data correspond to triplicates with means ± standard error of relative luciferase activity. A two-way ANOVA test was used, $****P < 0.0001$, $***P < 0.001$, $*P < 0.05$.

 © 2017 The Authors
