# Supplementary material for: A Pseudomonas aeruginosa TIR effector mediates immune evasion by targeting UBAP1 and TLR adaptors
Source: EMBO J. 2017 May 8;36(13):1869–87. doi: 10.15252/embj.201695343 (PMC5494471; doi:10.15252/embj.201695343)
Supplement: Supplementary file 3 — Source Data for Expanded View and Appendix [file EMBJ-36-1869-s007.zip › Source_Data_for_Appendix_and_EV_Figures/SourceData_for_Appendix_figure2/SD_for_Appendix_FigureS2C.pdf]

Fig_2C

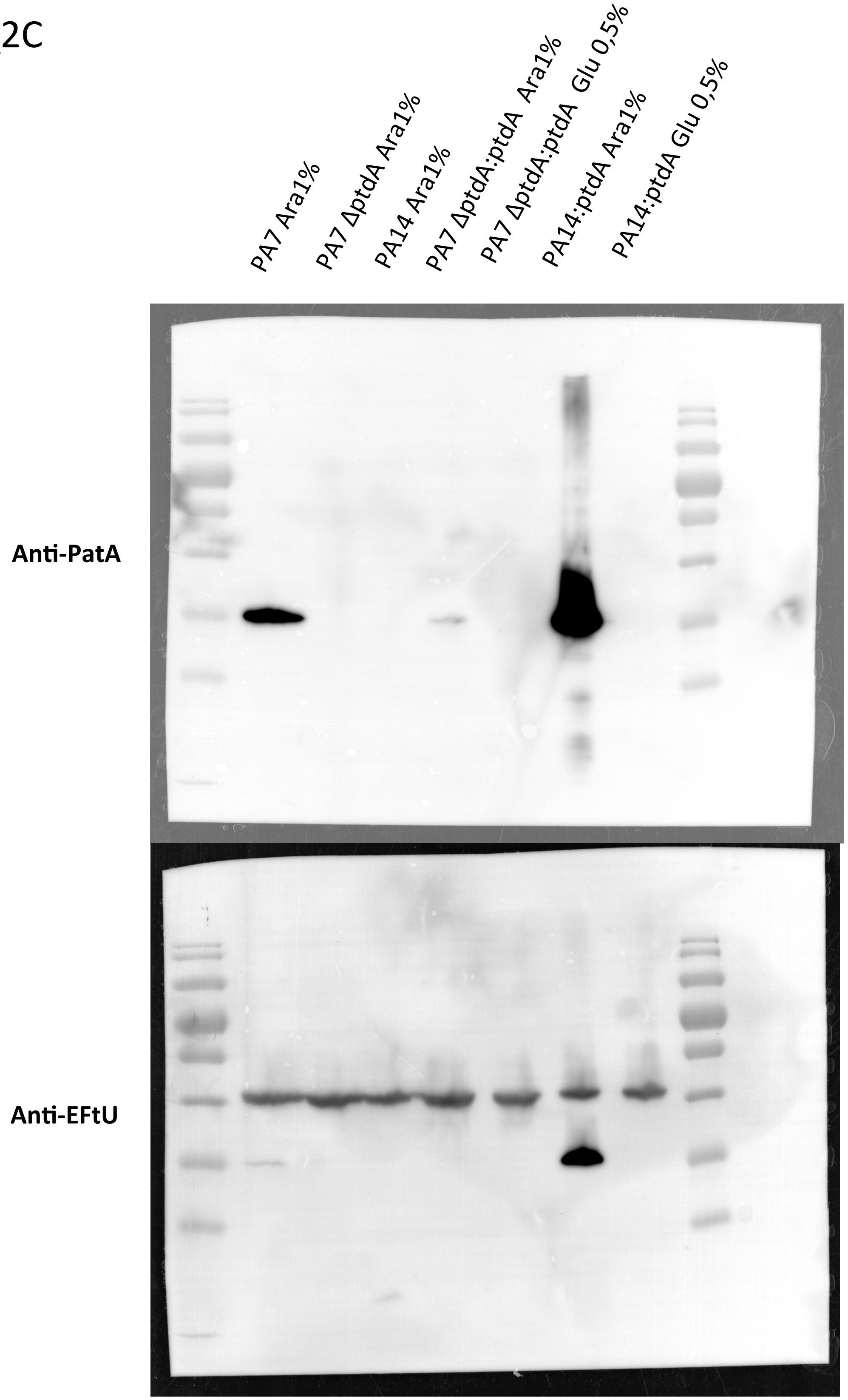
PA7 Ara1%
PA7 ΔptdA Ara1%
PA14 Ara1%
PA7 ΔptdA:ptdA Ara1%
PA7 ΔptdA:ptdA Glu 0,5%
PA14:ptdA Ara1%
PA14:ptdA Glu 0,5%
Anti-PatA
Anti-EFtU
