# Supplementary material for: A Pseudomonas aeruginosa TIR effector mediates immune evasion by targeting UBAP1 and TLR adaptors
Source: EMBO J. 2017 May 8;36(13):1869–87. doi: 10.15252/embj.201695343 (PMC5494471; doi:10.15252/embj.201695343)

FigEV4B

GFP+Myc-MyD88
GFPPumA$_{137\text{-}303}$+Myc-MyD88

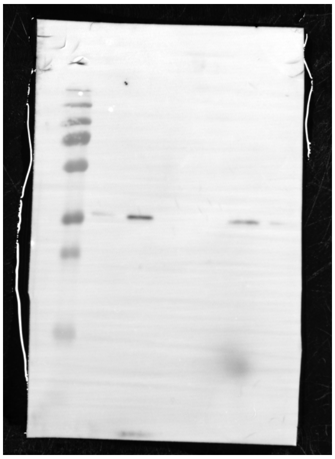

αMyc IP

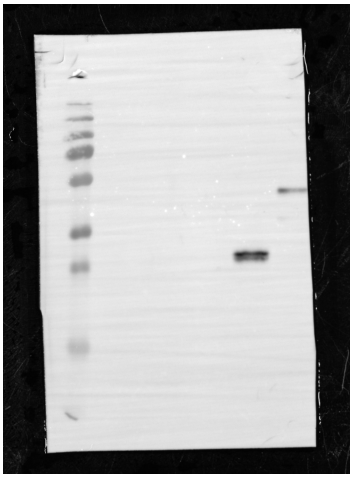

αGFP IP

GFP+Myc-MyD88
GFPPumA$_{137\text{-}303}$+Myc-MyD88

αMyc
INPUT

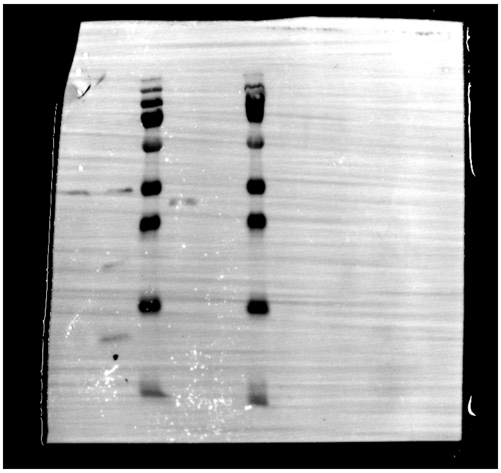

αGFP
INPUT

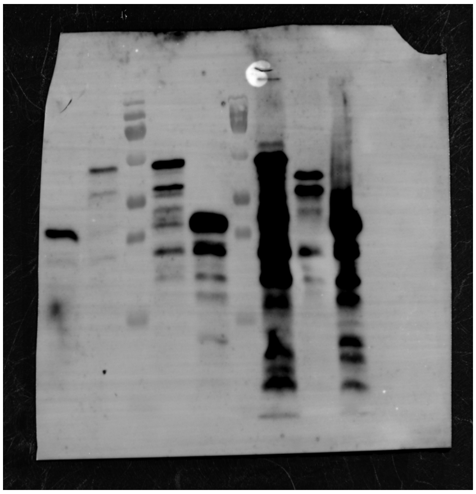

Supplement: Supplementary file 3 — Source Data for Expanded View and Appendix [file EMBJ-36-1869-s007.zip › Source_Data_for_Appendix_and_EV_Figures/SourceData_for_FigureEV4/SD_for_FigEV4B.pdf]
