# Supplementary figures and images for: A Pseudomonas aeruginosa TIR effector mediates immune evasion by targeting UBAP1 and TLR adaptors
Source: EMBO J. 2017 May 8;36(13):1869–87. doi: 10.15252/embj.201695343 (PMC5494471; doi:10.15252/embj.201695343)

Fig\_S2B

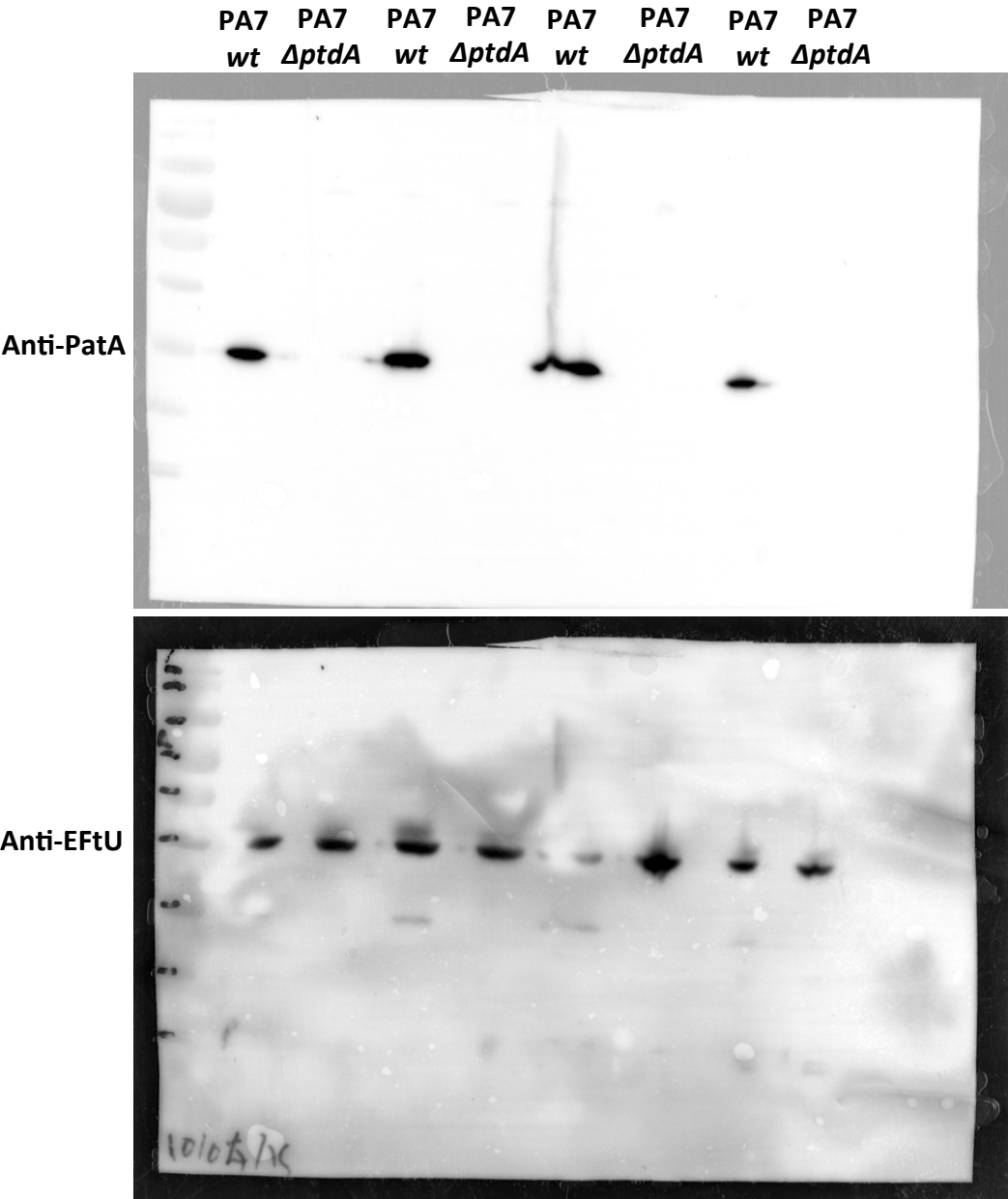

Supplement: Supplementary file 3 — Source Data for Expanded View and Appendix [file EMBJ-36-1869-s007.zip › Source_Data_for_Appendix_and_EV_Figures/SourceData_for_Appendix_figure2/SD_for_Appendix_FigureS2B.pdf]

Fig\_S4A

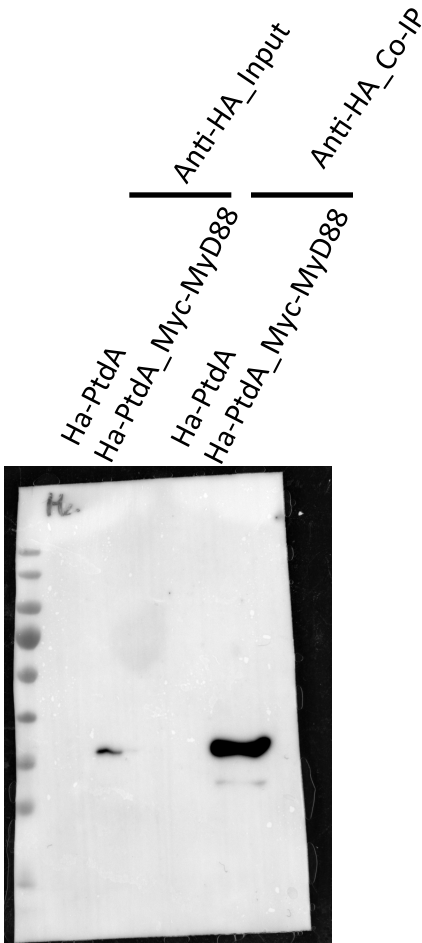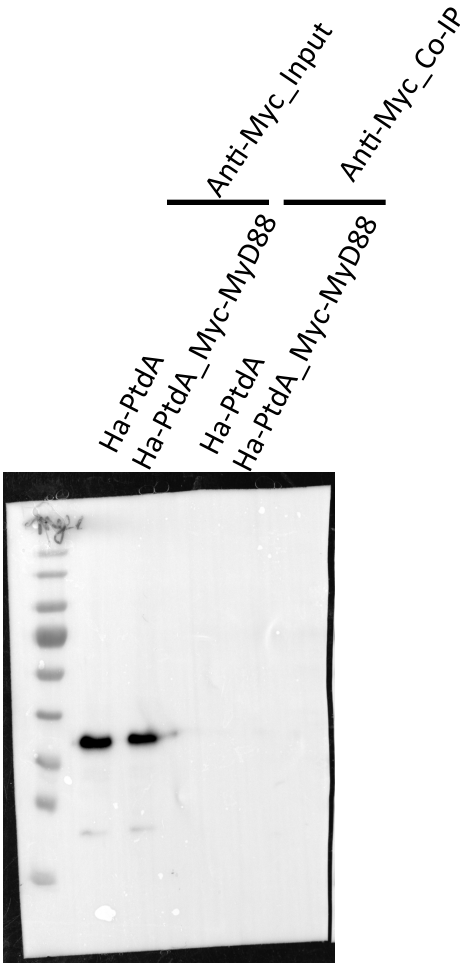

Supplement: Supplementary file 3 — Source Data for Expanded View and Appendix [file EMBJ-36-1869-s007.zip › Source_Data_for_Appendix_and_EV_Figures/SourceData_for_Appendix_Figure4/SD_for_Appendix_FigureS4A.pdf]

Fig\_S4B

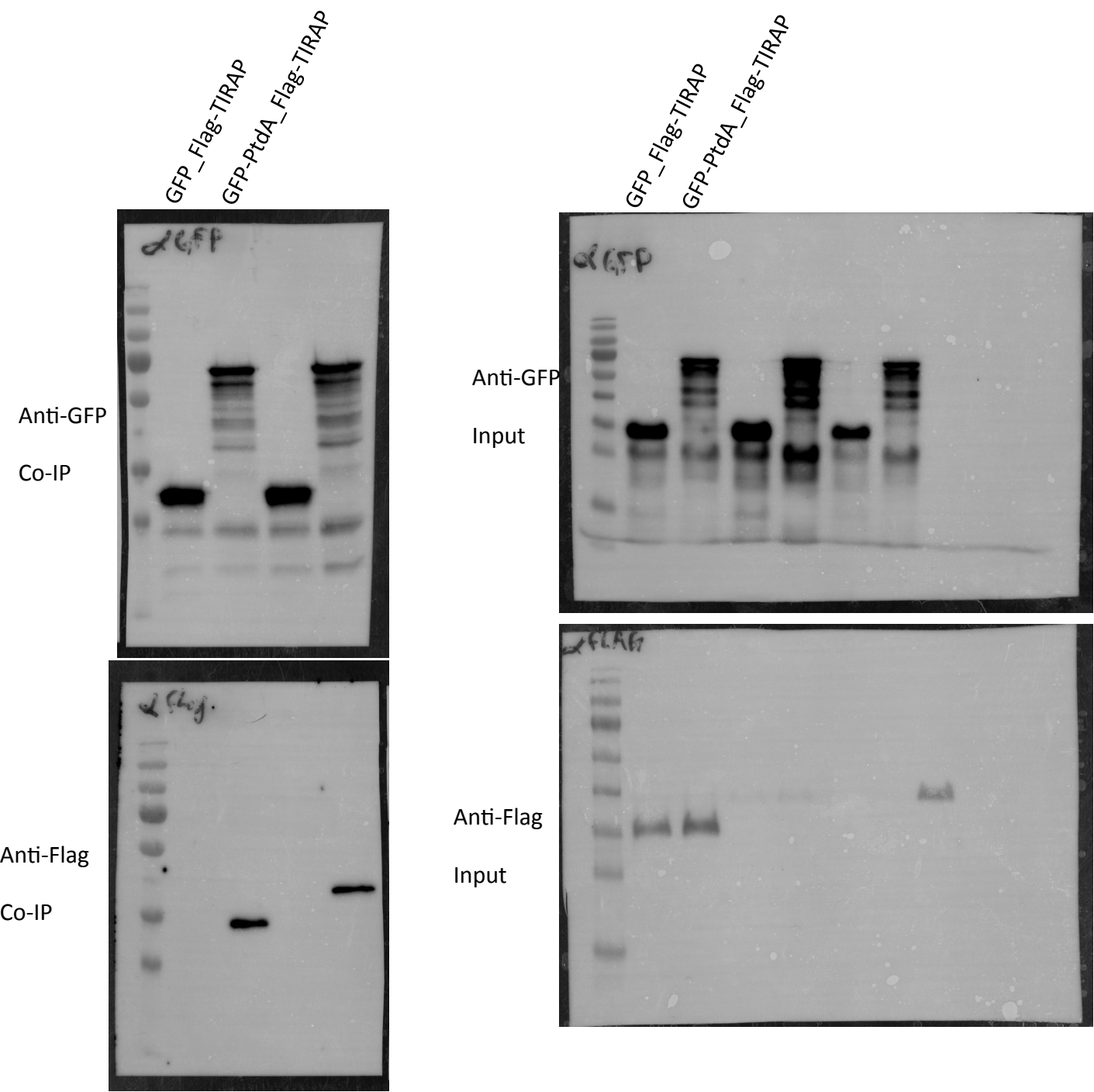

Supplement: Supplementary file 3 — Source Data for Expanded View and Appendix [file EMBJ-36-1869-s007.zip › Source_Data_for_Appendix_and_EV_Figures/SourceData_for_Appendix_Figure4/SD_for_Appendix_FigureS4B.pdf]

Fig\_S4C

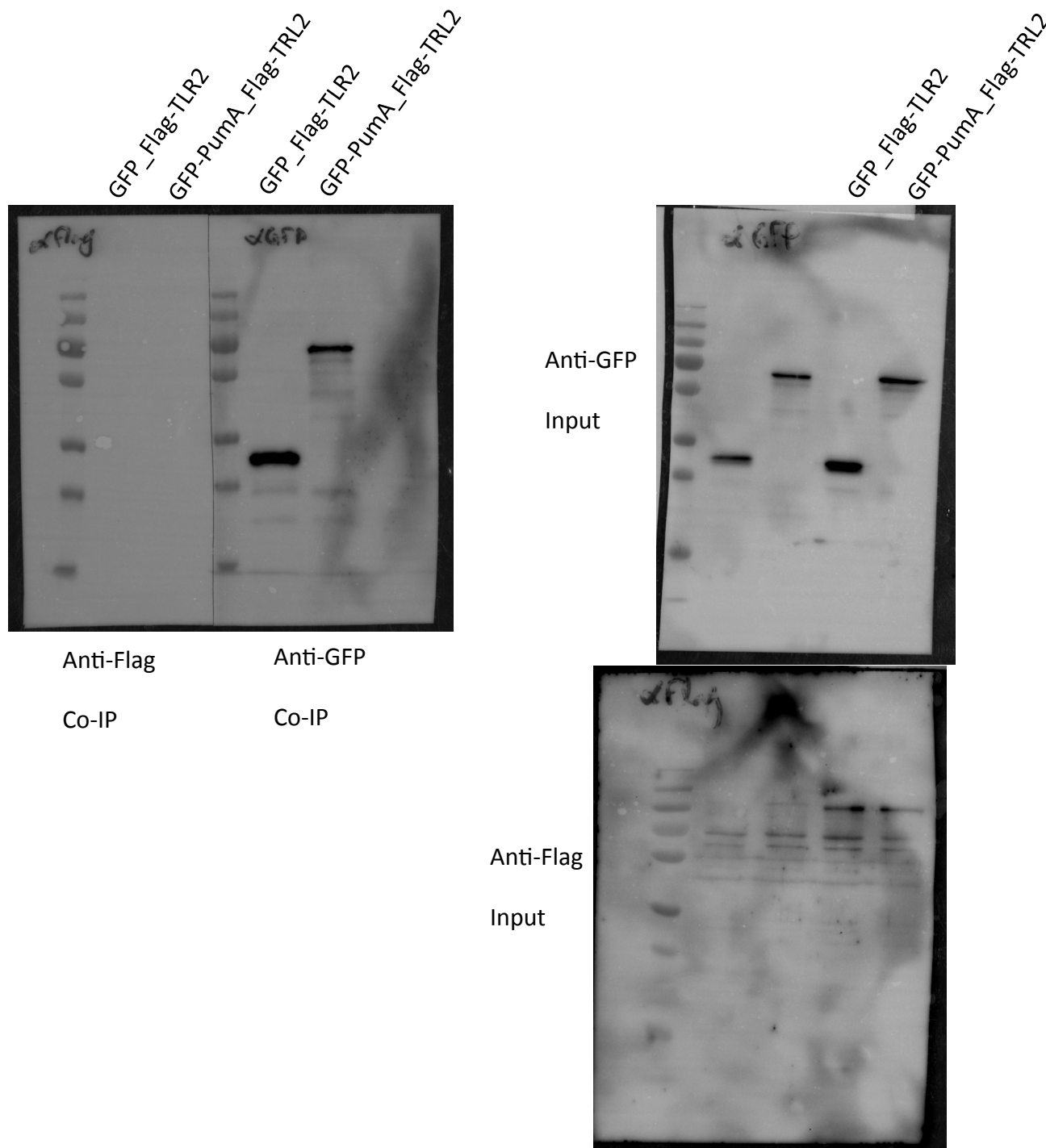

Supplement: Supplementary file 3 — Source Data for Expanded View and Appendix [file EMBJ-36-1869-s007.zip › Source_Data_for_Appendix_and_EV_Figures/SourceData_for_Appendix_Figure4/SD_for_Appendix_FigureS4C.pdf]

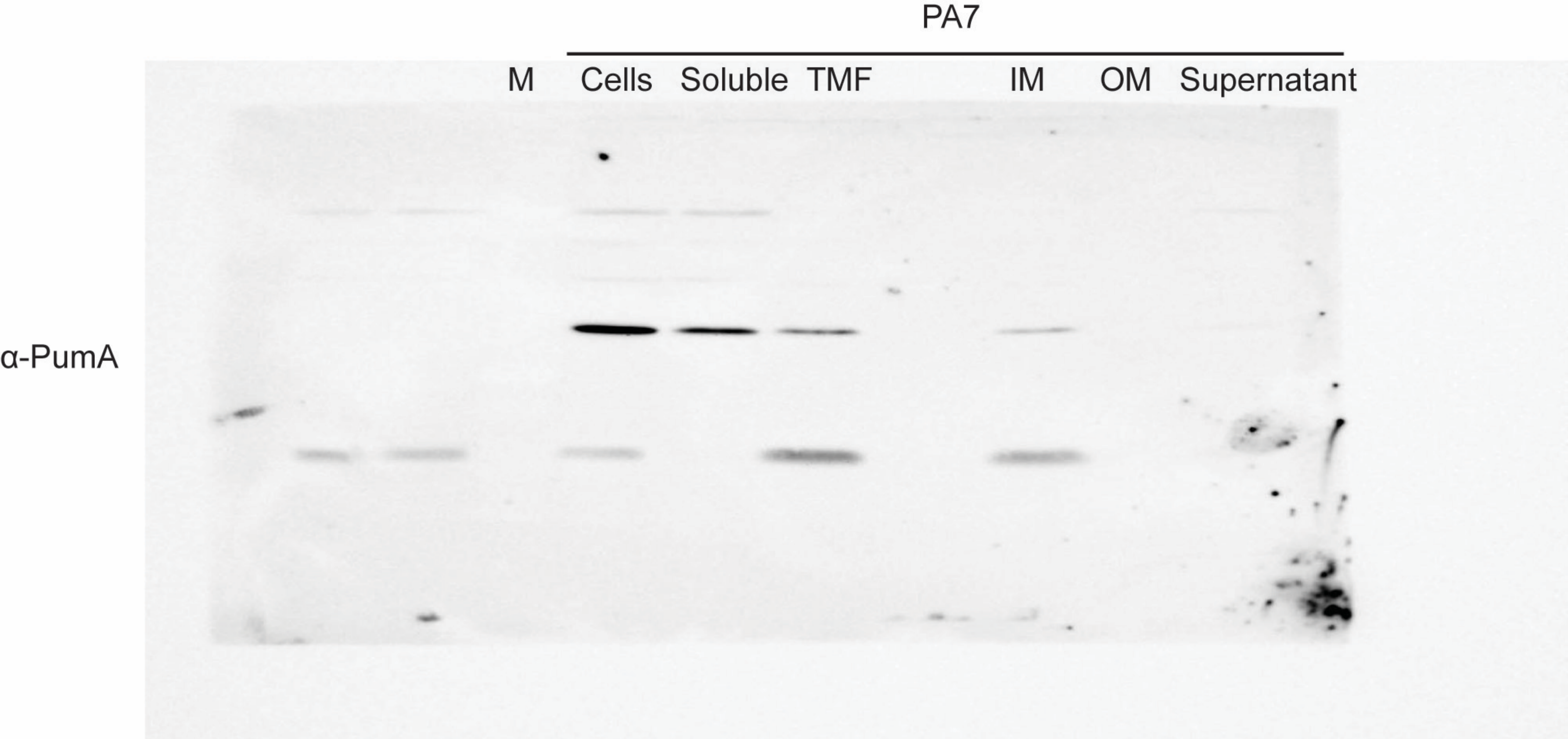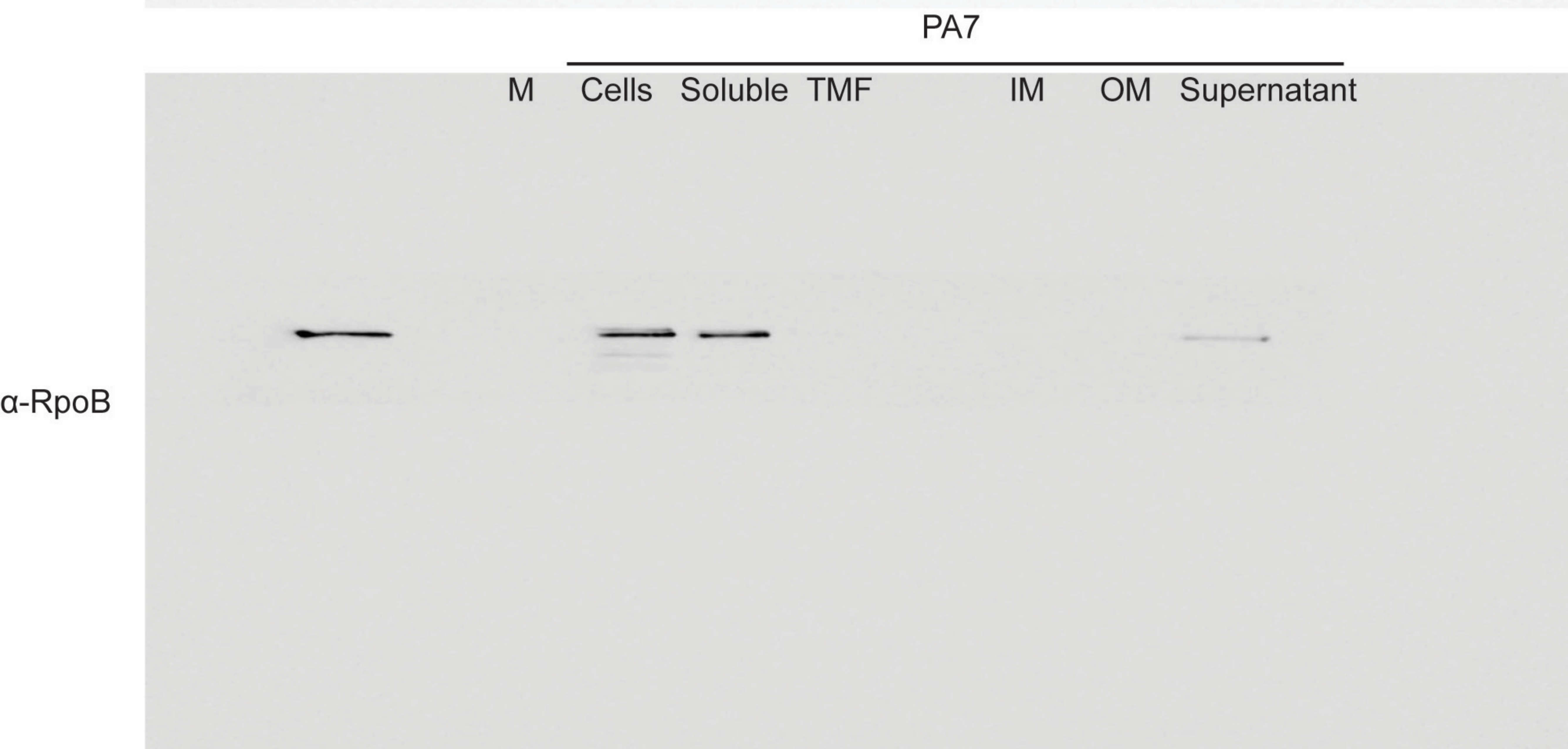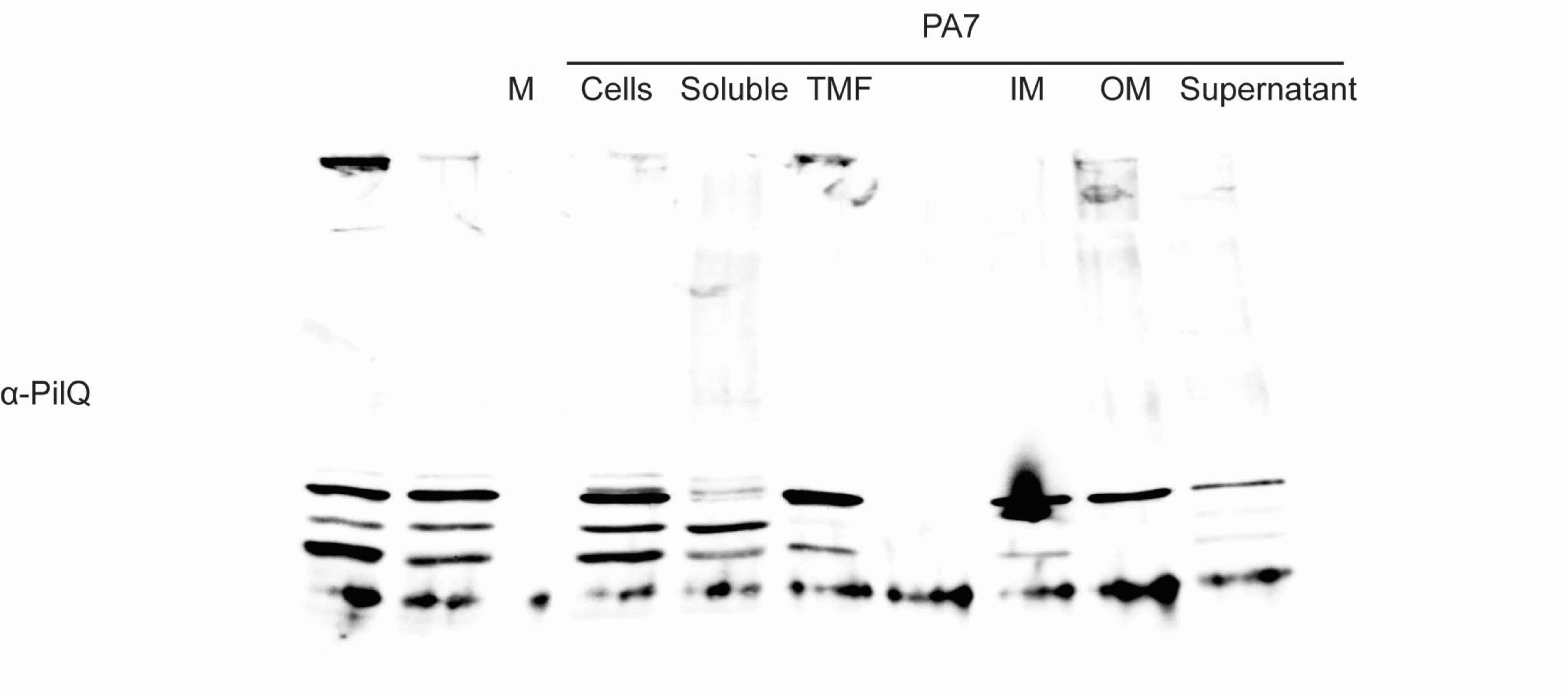

Supplement: Supplementary file 3 — Source Data for Expanded View and Appendix [file EMBJ-36-1869-s007.zip › Source_Data_for_Appendix_and_EV_Figures/SourceData_for_Appendix_FigureS3/SD_for_Appendix_FigureS3A.pdf]

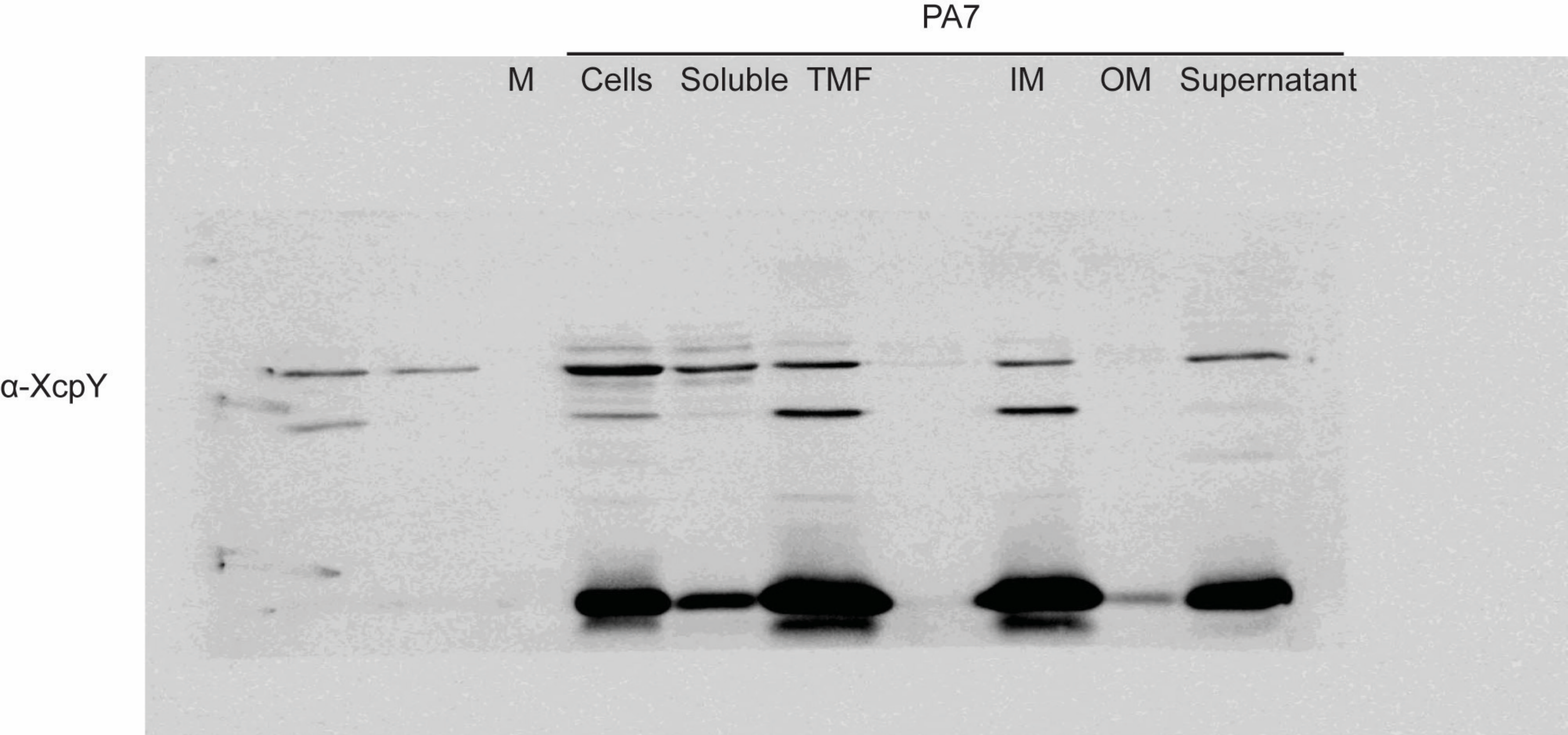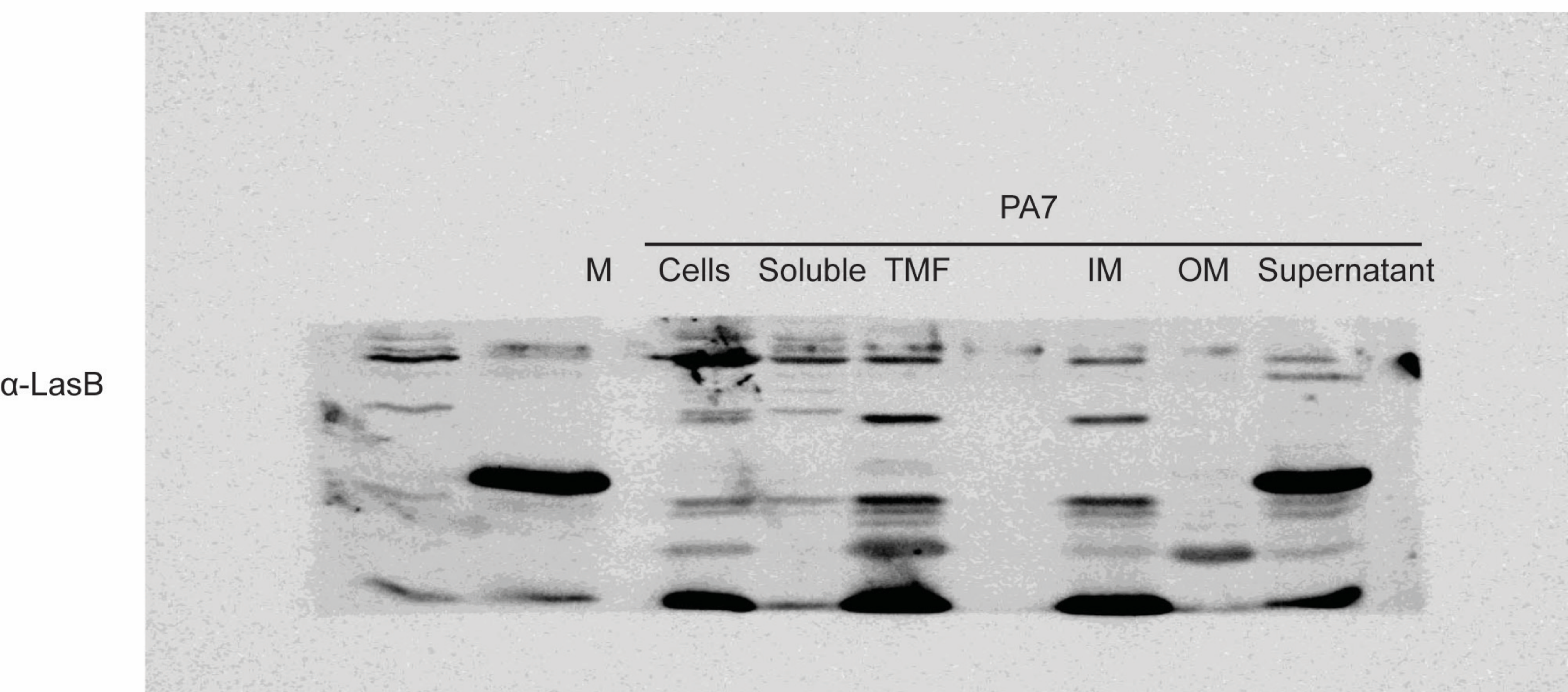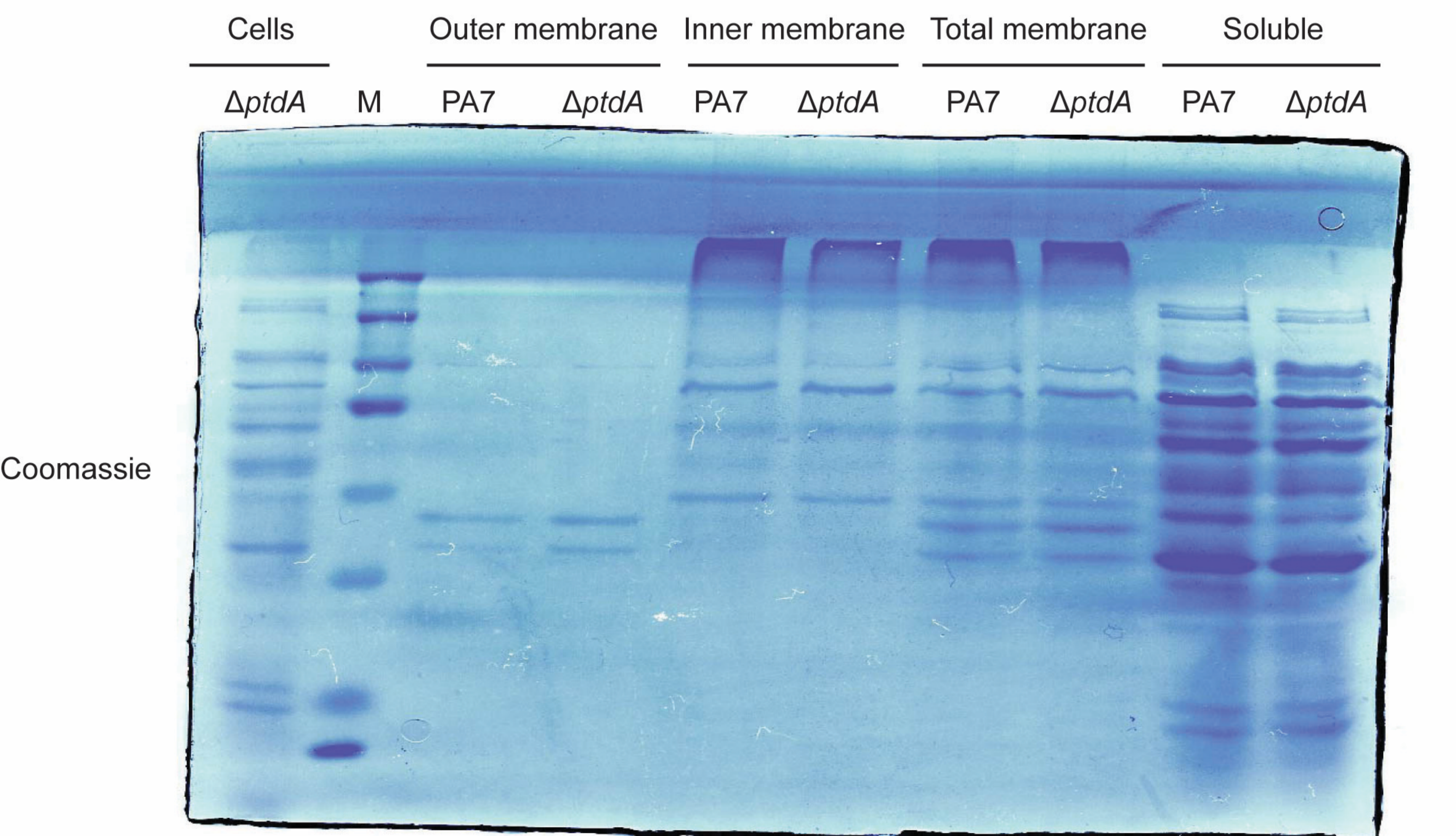

Supplement: Supplementary file 3 — Source Data for Expanded View and Appendix [file EMBJ-36-1869-s007.zip › Source_Data_for_Appendix_and_EV_Figures/SourceData_for_Appendix_FigureS3/SD_for_Appendix_FigureS3A2.pdf]

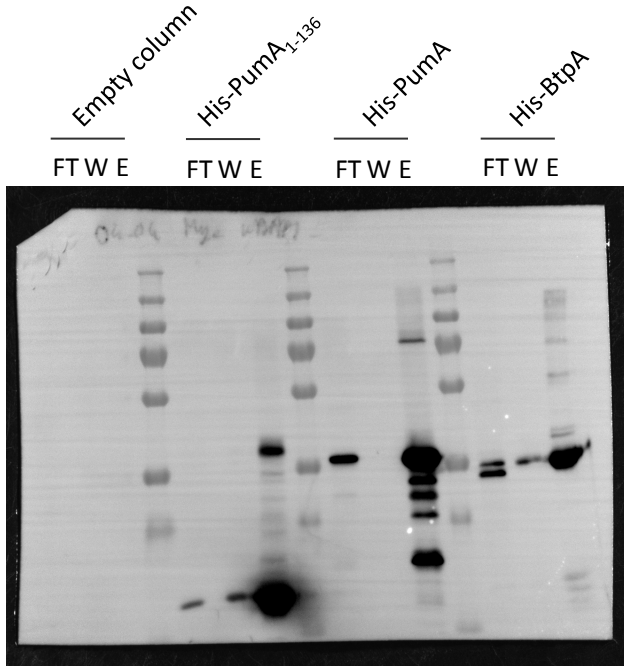

FigS5A :  $\alpha$ V5 Myc-UBAP1

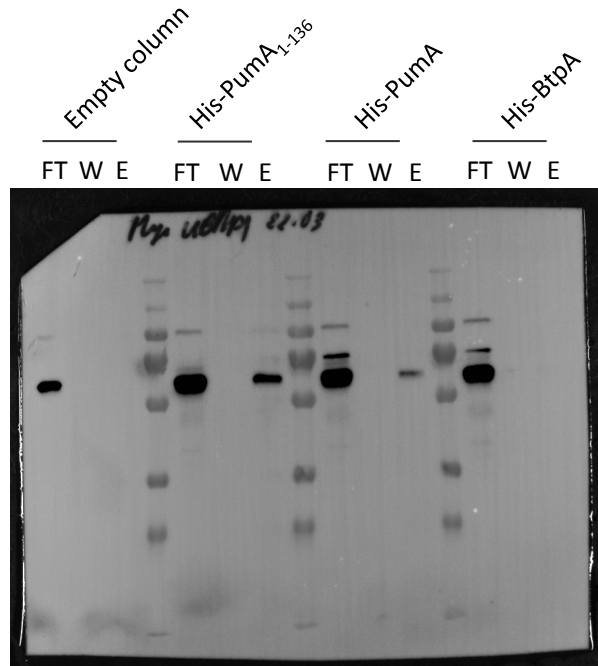

FigS5A :  $\alpha$ UBAP1 Myc-UBAP1

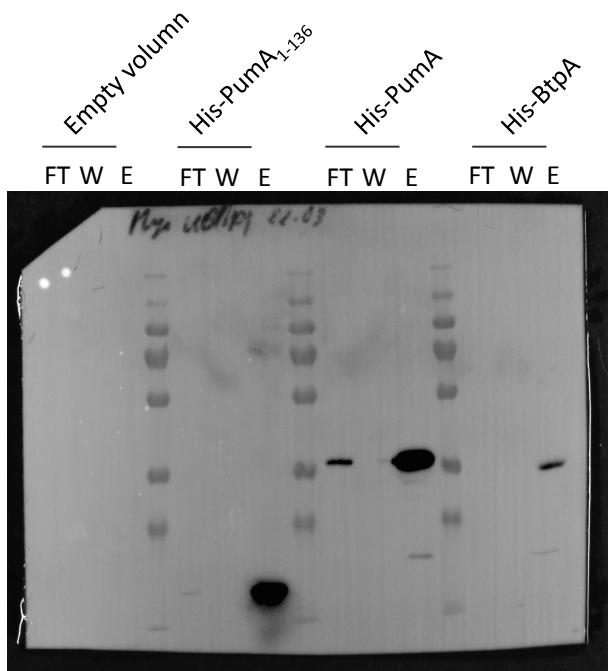

FigS5A :  $\alpha$ His Myc-UBAP1

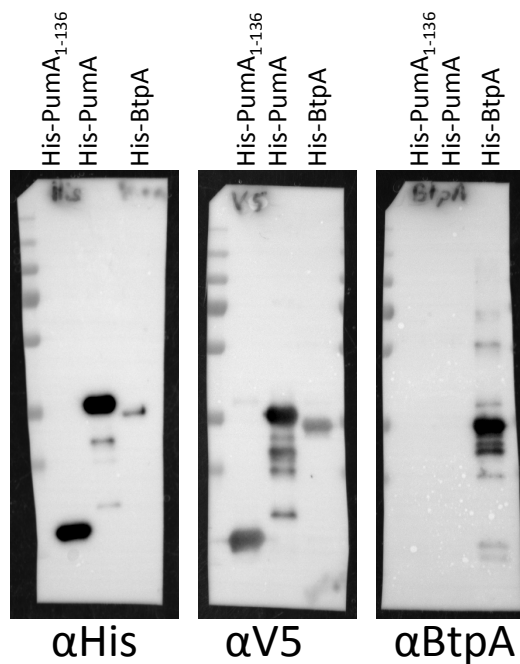

FigS5B

Supplement: Supplementary file 3 — Source Data for Expanded View and Appendix [file EMBJ-36-1869-s007.zip › Source_Data_for_Appendix_and_EV_Figures/SourceData_for_Appendix_FigureS5/SD_for_Appendix_FigureS5A.pdf]

Fig\_S5D

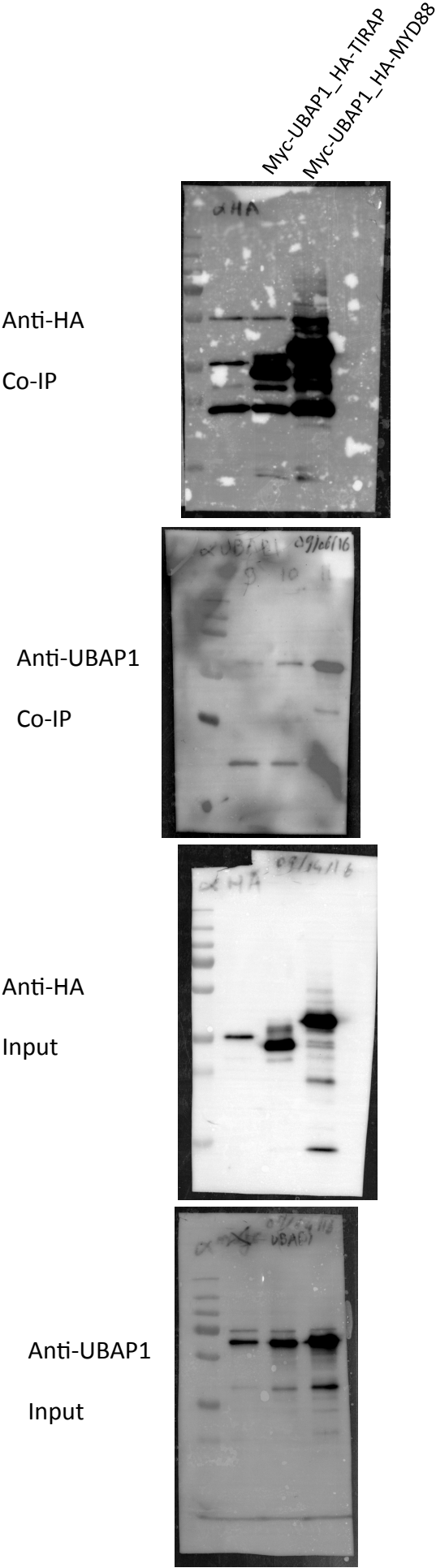

Supplement: Supplementary file 3 — Source Data for Expanded View and Appendix [file EMBJ-36-1869-s007.zip › Source_Data_for_Appendix_and_EV_Figures/SourceData_for_Appendix_FigureS5/SD_for_Appendix_FigureS5D.pdf]

Fig\_S5E

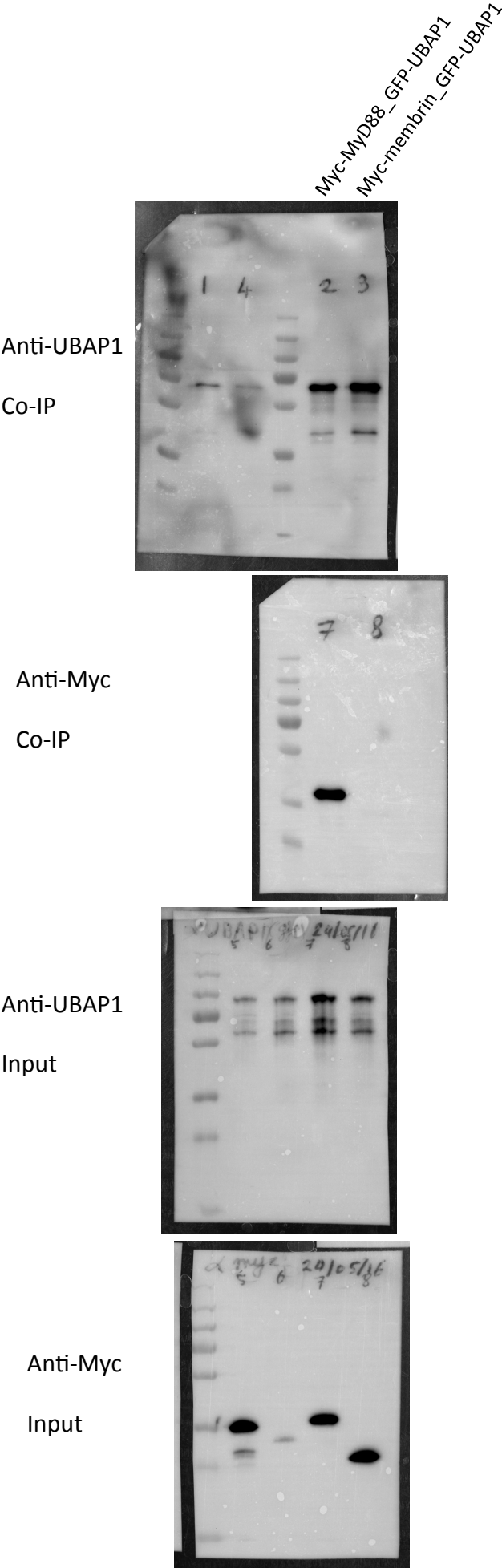

Supplement: Supplementary file 3 — Source Data for Expanded View and Appendix [file EMBJ-36-1869-s007.zip › Source_Data_for_Appendix_and_EV_Figures/SourceData_for_Appendix_FigureS5/SD_for_Appendix_FigureS5E.pdf]

GFP+Myc-UBAP1  
GFPPumA<sup>137-303</sup>+Myc-UBAP1

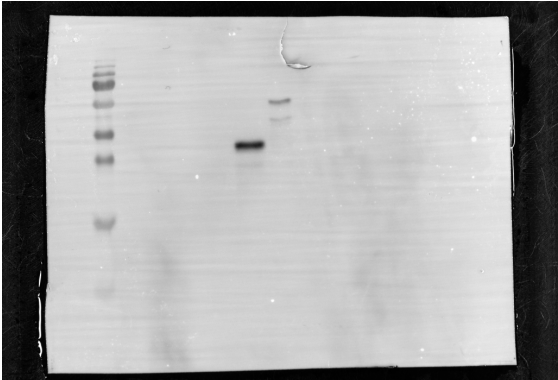

$\alpha$ GFP IP

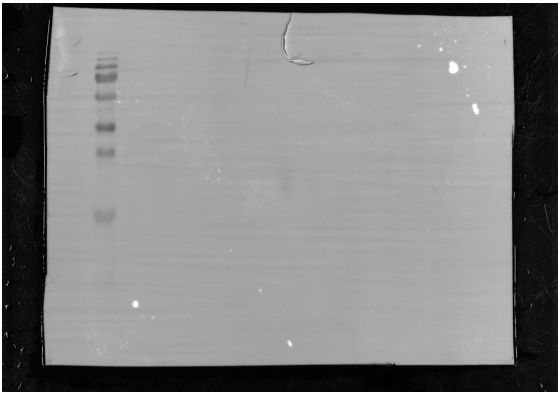

$\alpha$ Myc IP

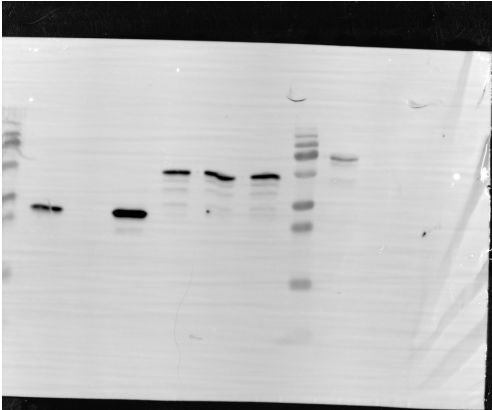

$\alpha$ GFP INPUT

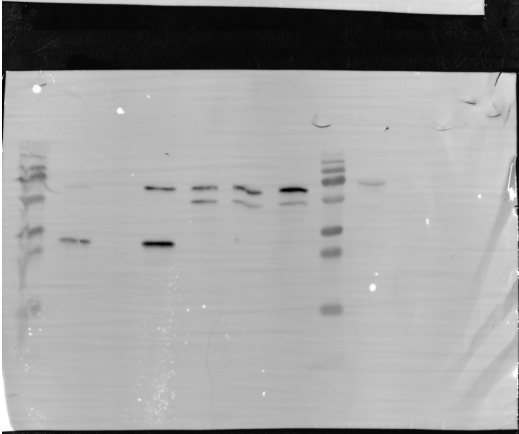

$\alpha$ myc INPUT

Supplement: Supplementary file 3 — Source Data for Expanded View and Appendix [file EMBJ-36-1869-s007.zip › Source_Data_for_Appendix_and_EV_Figures/SourceData_for_FigureEV4/SD_for_FigEV4D.pdf]

Fig\_4C

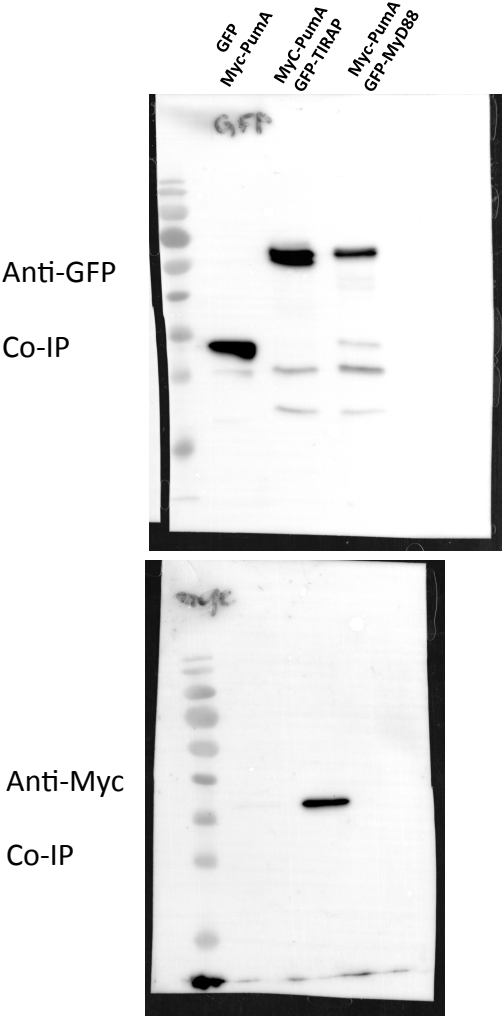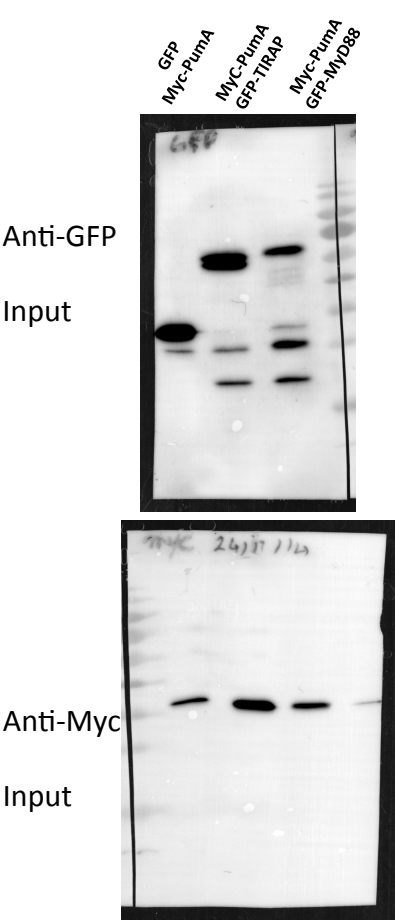

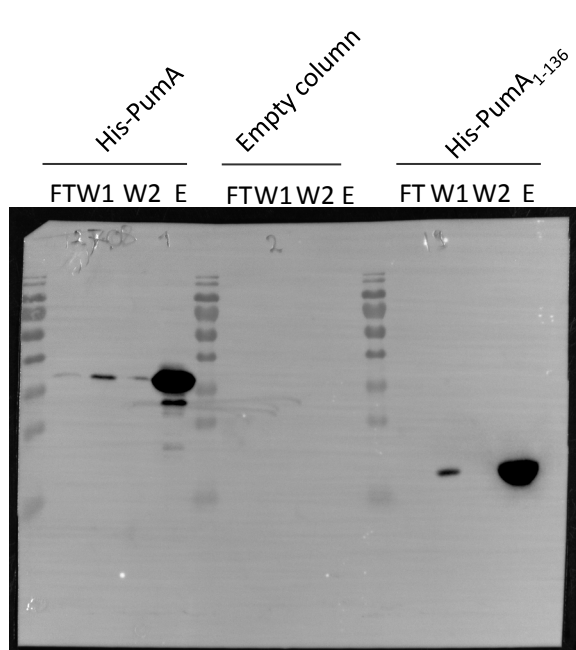

Fig4D : αHis HA-TIRAP

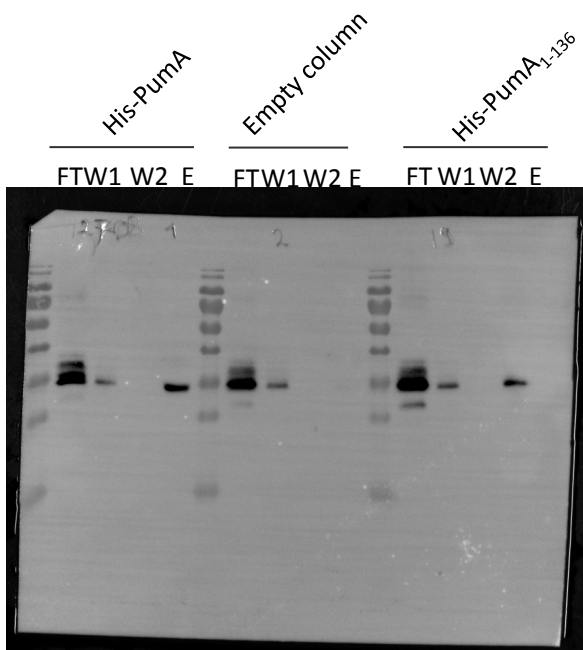

Fig4D : αHA HA-TIRAP

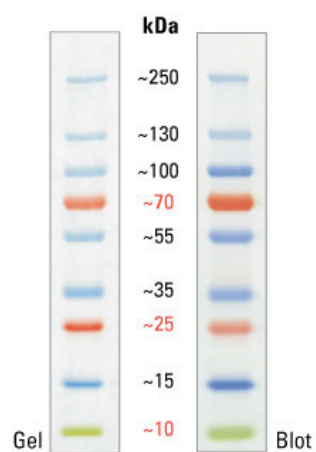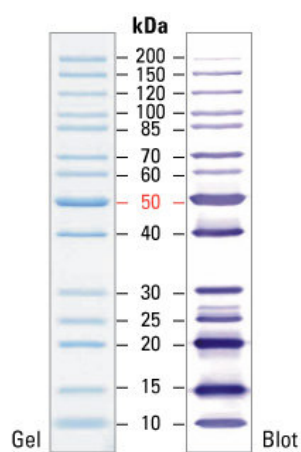

Supplement: Supplementary file 5 — Source Data for Figure 4 [file EMBJ-36-1869-s003.pdf]

Fig\_5B

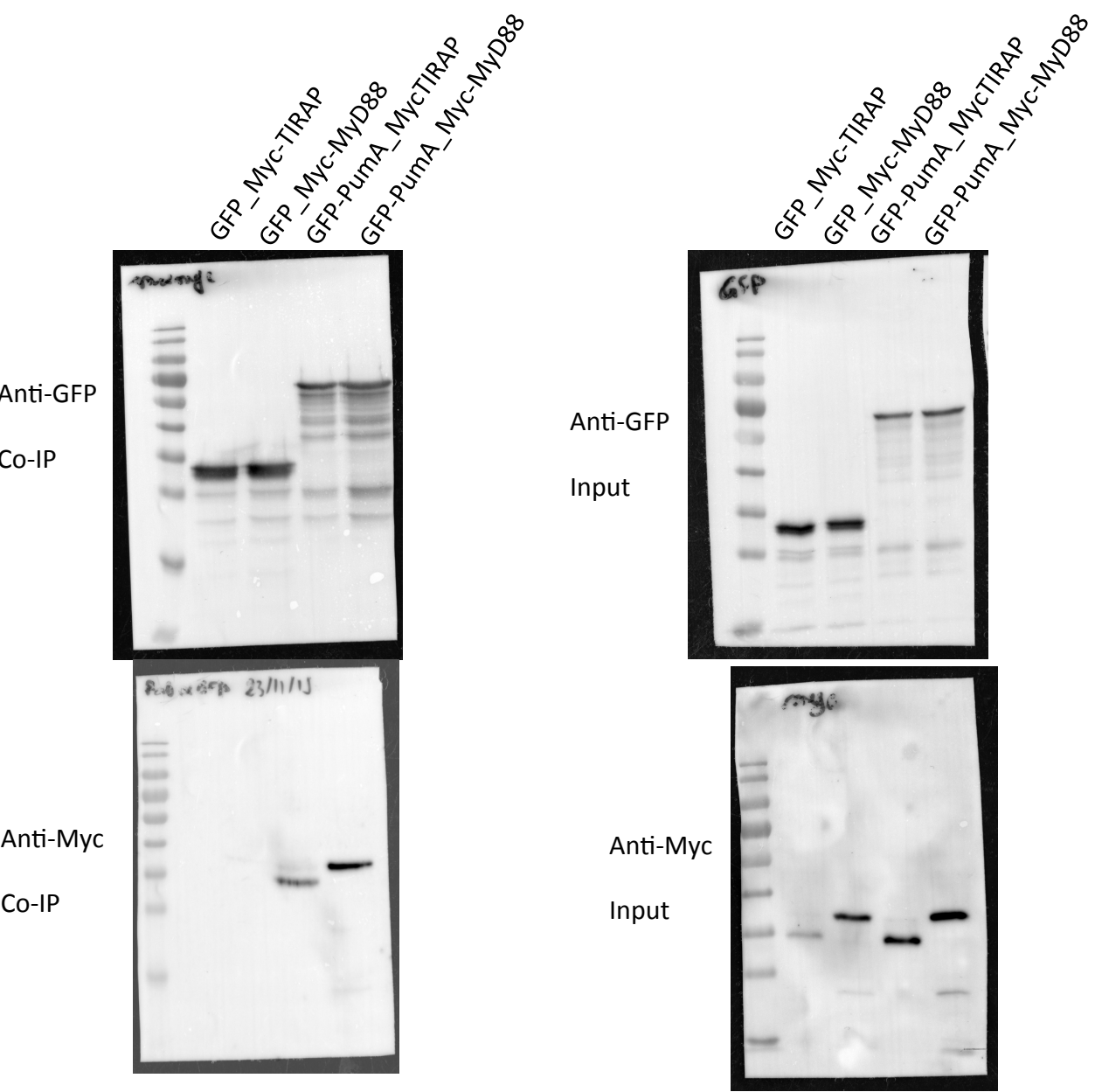

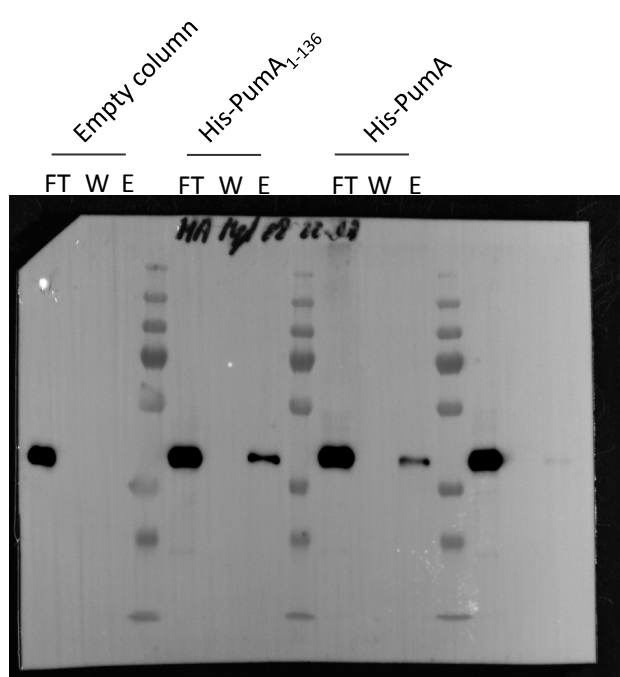

Fig5A :  $\alpha$ HA HA-Myd88

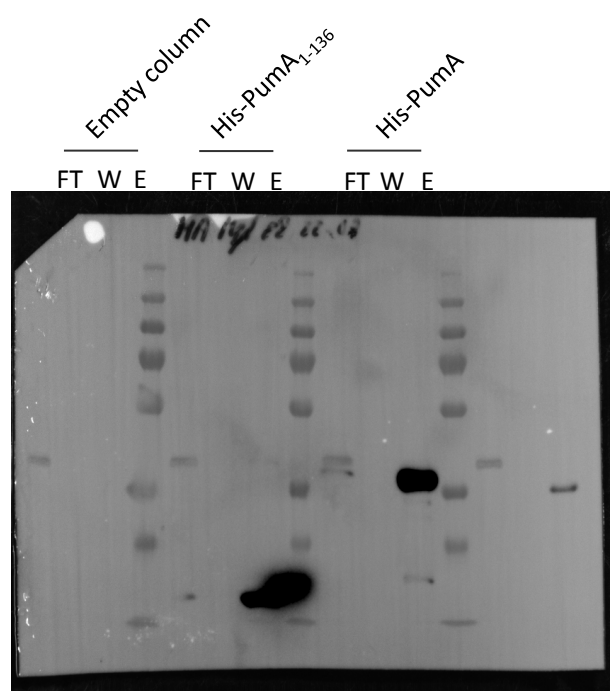

Fig5A :  $\alpha$ His HA-Myd88

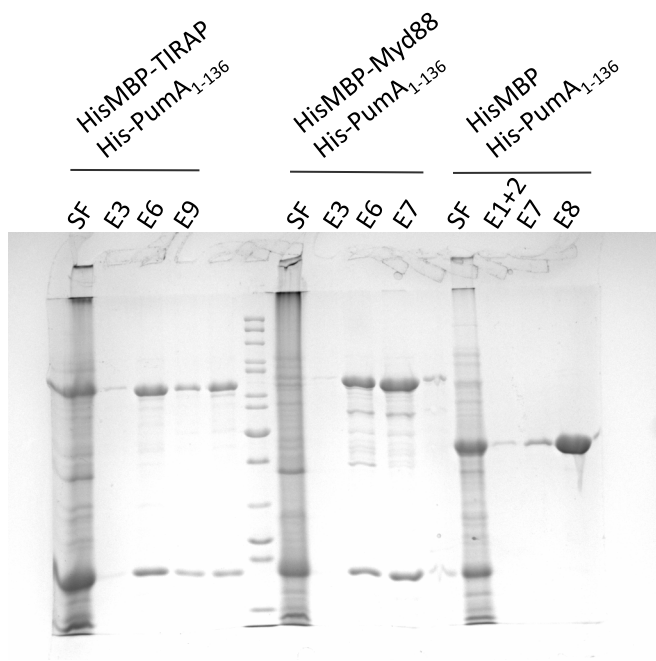

Fig5 D-E-C

Supplement: Supplementary file 6 — Source Data for Figure 5 [file EMBJ-36-1869-s004.pdf]

Fig\_6A

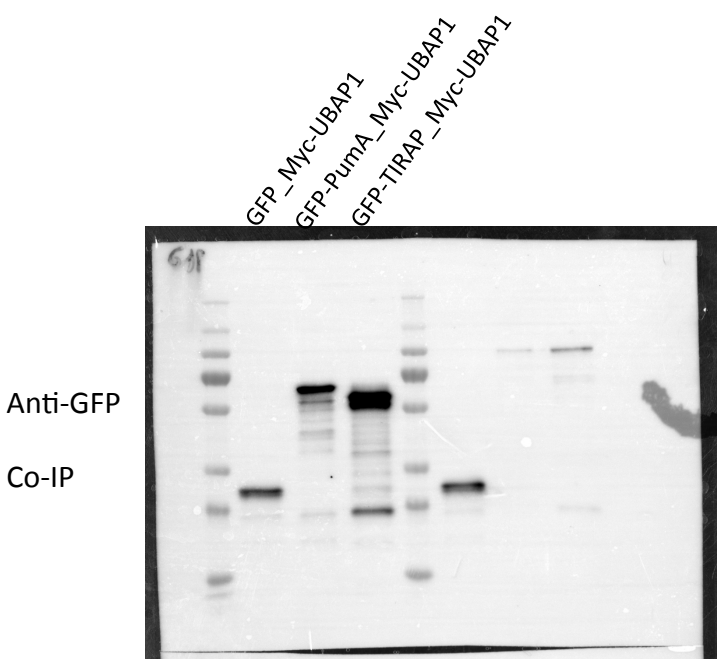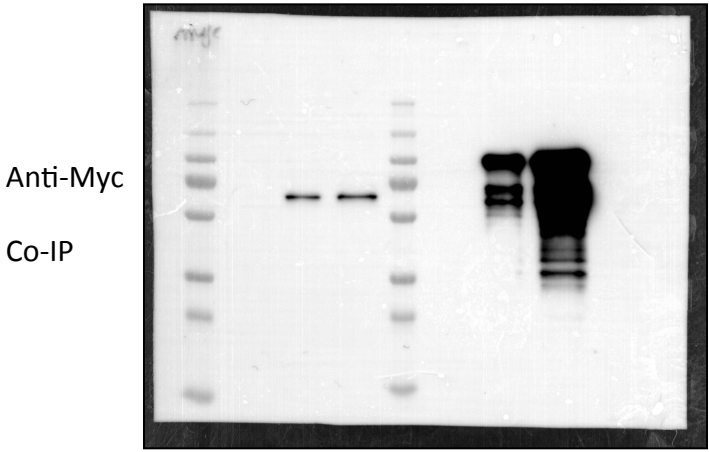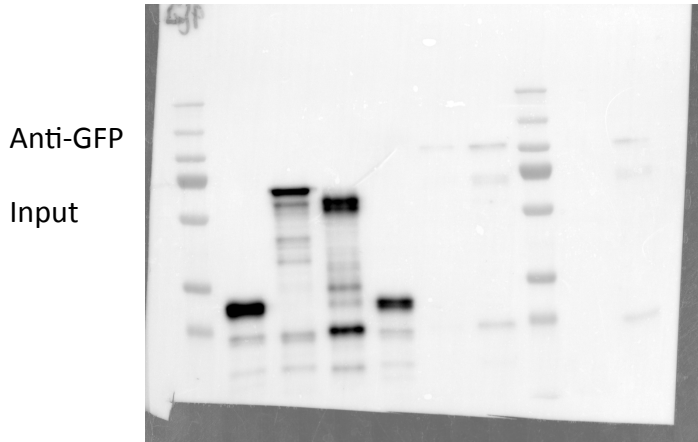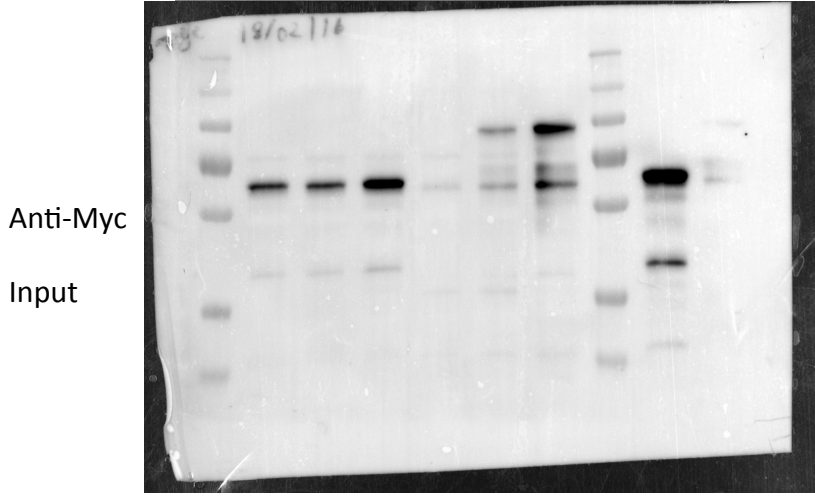

Fig\_6B

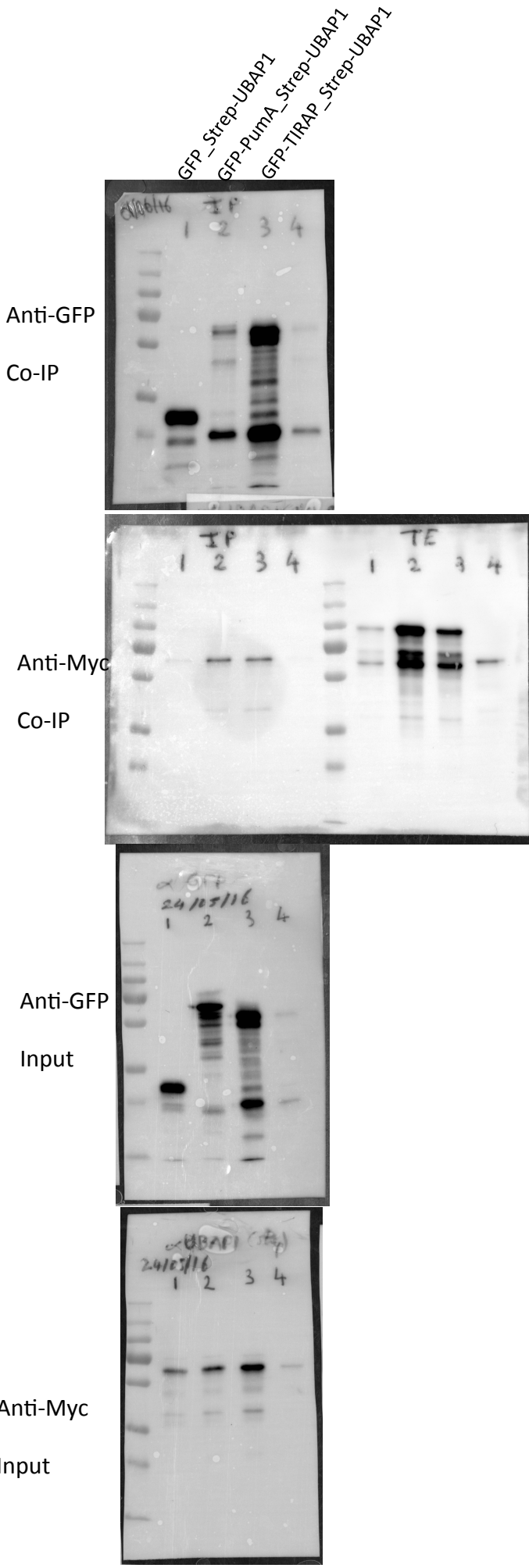

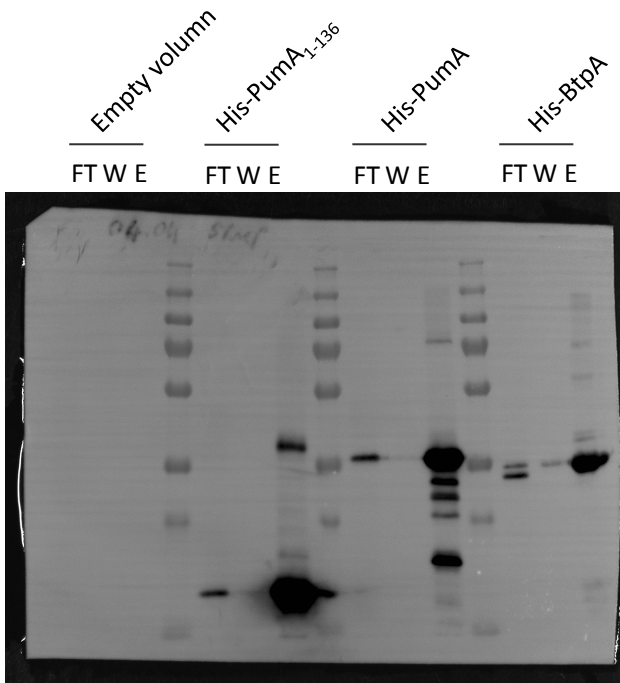

Fig6C :  $\alpha$ V5 Strep-UBAP1

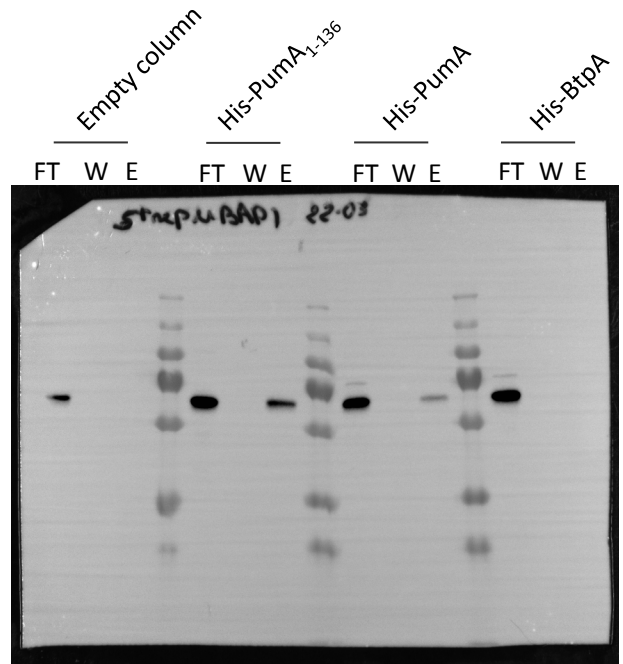

Fig6C :  $\alpha$ UBAP1 Strep-UBAP1

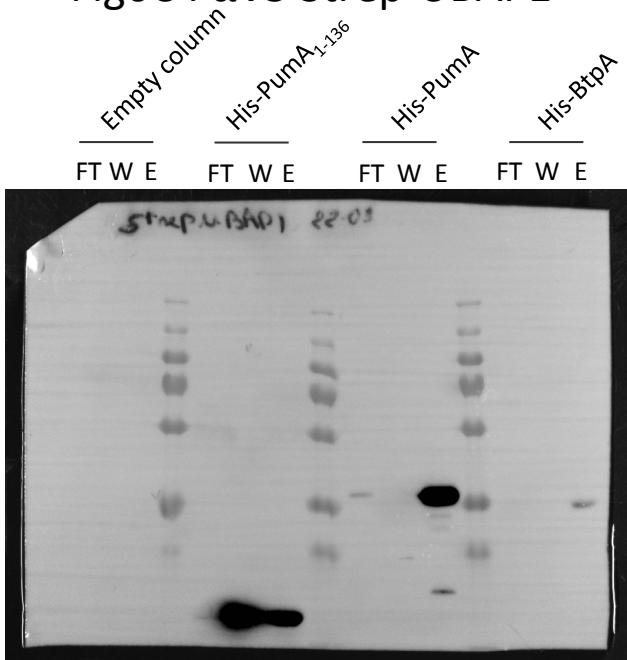

Fig6C :  $\alpha$ His Strep-UBAP1

Fig\_6D, E and F

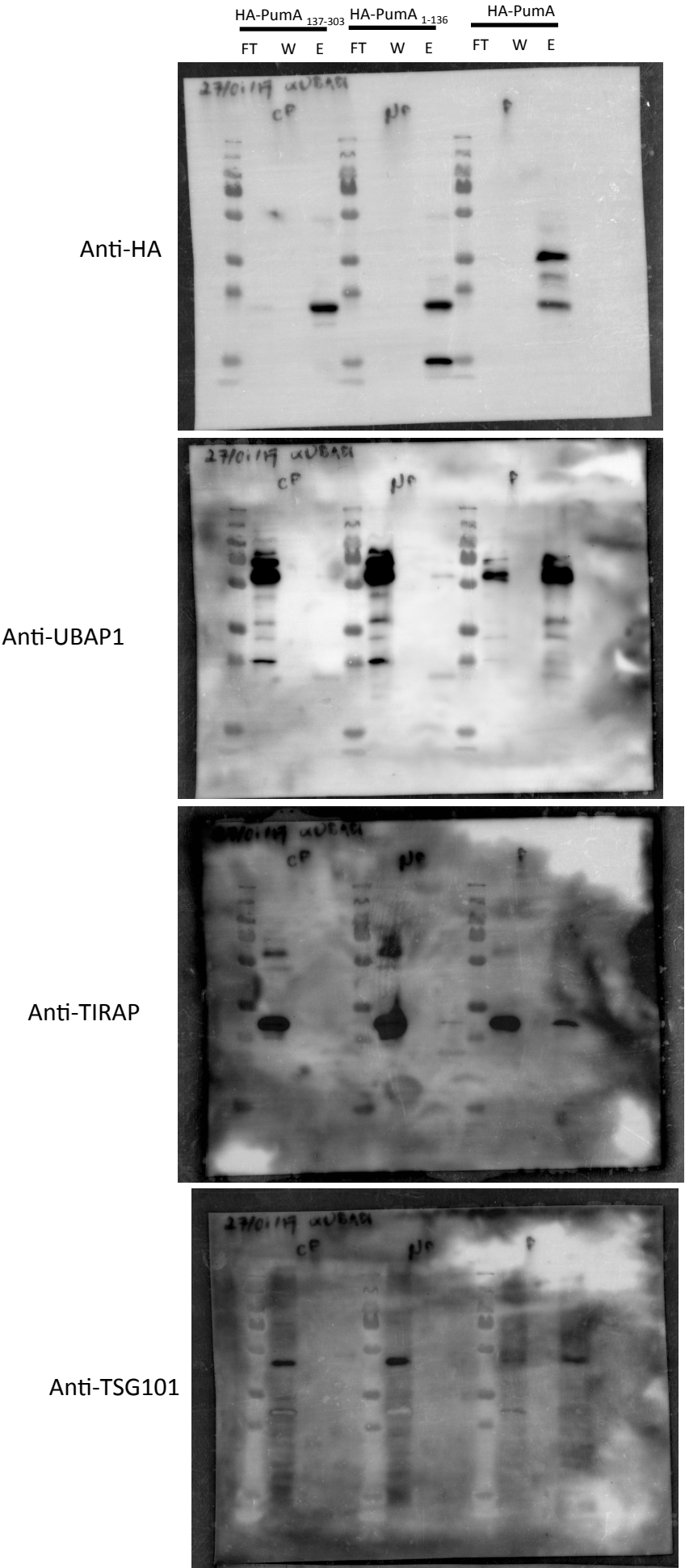

Supplement: Supplementary file 7 — Source Data for Figure 6 [file EMBJ-36-1869-s005.pdf]

Fig\_Fig7D and E

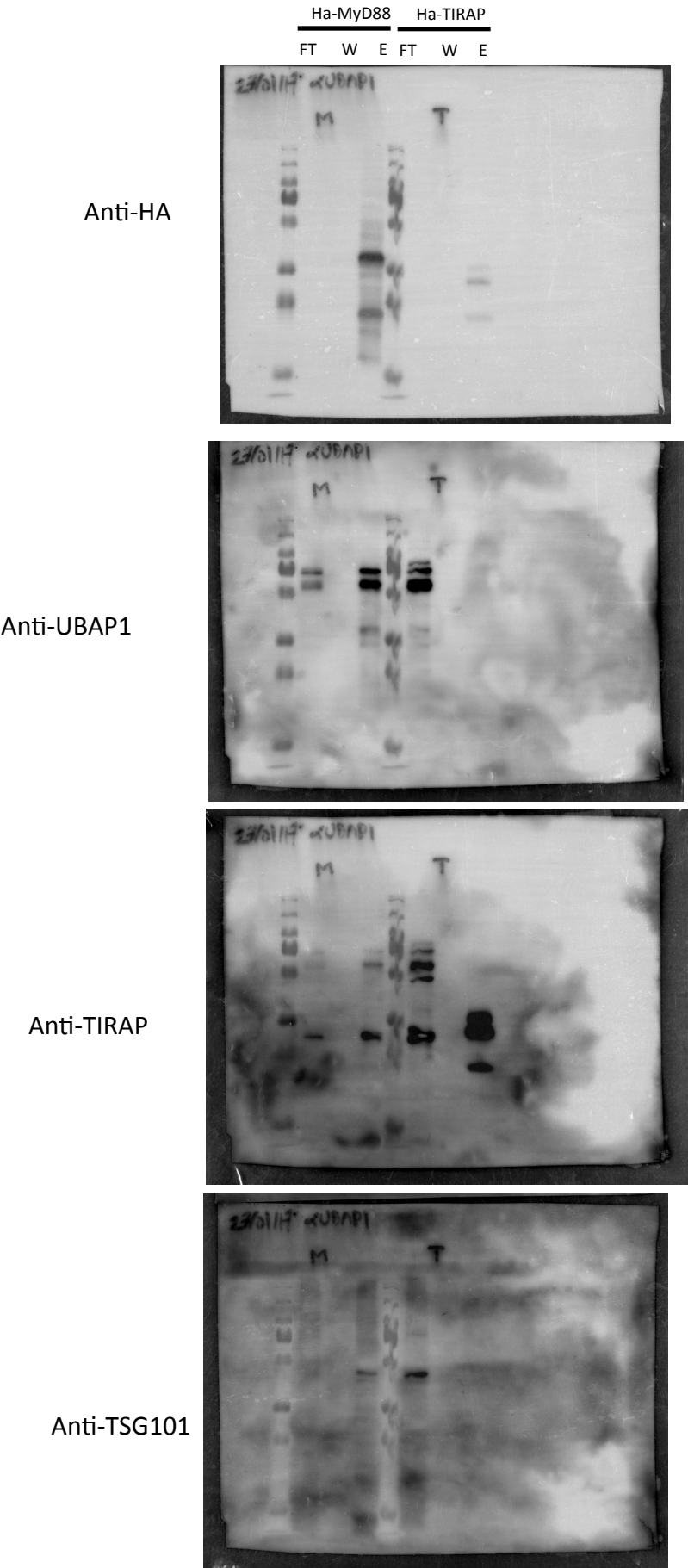

Fig\_Fig7F

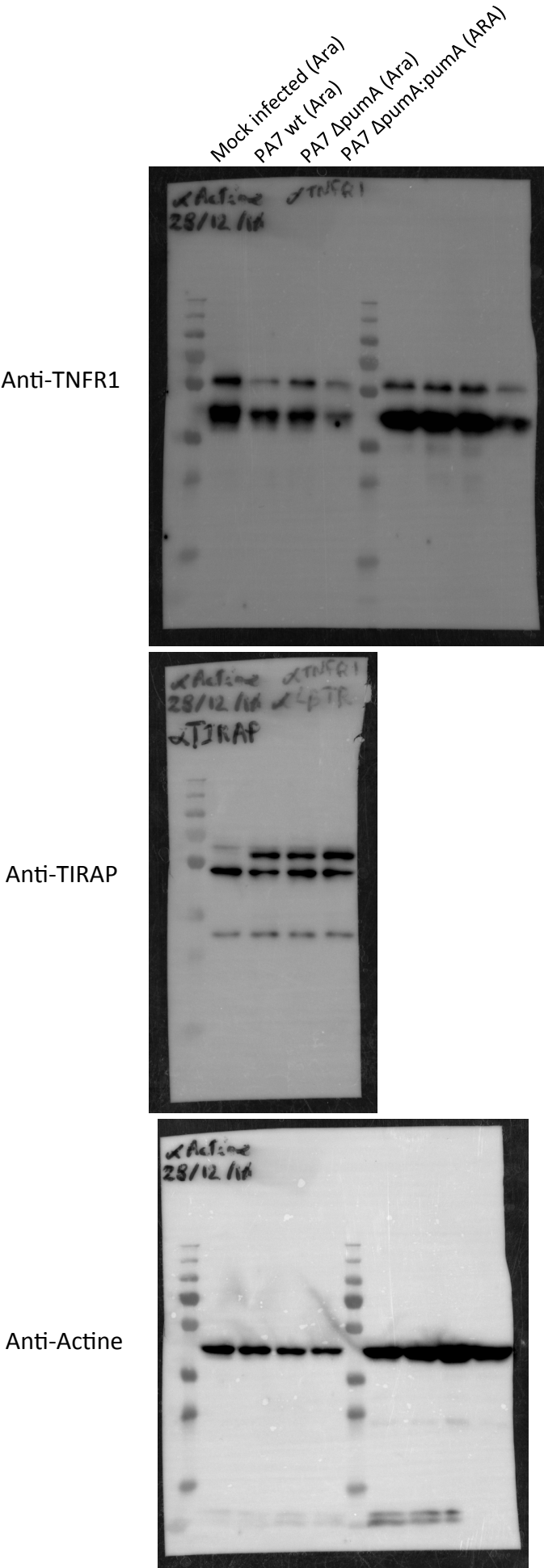

Supplement: Supplementary file 8 — Source Data for Figure 7 [file EMBJ-36-1869-s006.pdf]
